# Systematics of the new genus *Spinosuncus* Chen, Zhang & Li with descriptions of four new species (Lepidoptera, Crambidae, Pyraustinae)

**DOI:** 10.3897/zookeys.799.23925

**Published:** 2018-11-28

**Authors:** Kai Chen, Dandan Zhang, Houhun Li

**Affiliations:** 1 State Key Laboratory of Biocontrol/The Museum of Biology, School of Life Sciences, Sun Yat-sen University, Guangzhou, Guangdong 510275, China Sun Yat-sen University Guangzhou China; 2 College of Life Sciences, Nankai University, Tianjin 300071, China Nankai University Tianjin China

**Keywords:** *
Aglaops
*, China, molecular phylogeny, new combinations, *
Ostrinia
*, *
Paratalanta
*, *
Placosaris
*, *
Pseudebulea
*, *
Pseudopagyda
*, *
Thliptoceras
*

## Abstract

The new genus *Spinosuncus***gen. n.** is proposed for three known species, *S.contractalis* (Warren, 1896), **comb. n.**, *S.praepandalis* (Snellen, 1890), **comb. n.**, and *S.aureolalis* (Lederer, 1863), **comb. n.** and four new species, *S.rectacutus***sp. n.**, *S.brevacutus***sp. n.**, *S.curvisetaceus***sp. n.**, and *S.quadracutus***sp. n.** from the Oriental Region. An identification key is provided for all species. The habiti and genitalia of all species are figured. The monophyly of the genus is well supported by a phylogenetic analysis based on sequence data of the COI, 16S rRNA, and EF-1α genes. The potential sister groups of the new genus, the interspecific relationships and some intraspecific variations within the genus are discussed.

## Introduction

Pyraustinae is the third largest subfamily in the family Crambidae, containing 173 genera that include more than 1176 described species (Nuss et al. 2003–2018). The monophyly of the Pyraustinae is well supported by phylogenetic analyses based on both morphological characters and molecular data (Solis and Maes 2002, Regier et al. 2012). Based on specialized genitalic characters, e.g. the valva bearing sella and editum in the male genitalia and a rhomboidal signum in the female genitalia, pyraustine species are easily distinguished from members of its sister group, Spilomelinae (Regier et al. 2012). However, the taxa belonging to Pyraustinae*sensu stricto* still have not been all associated on a worldwide basis (Solis and Maes 2002), partly because they were for a long time placed into Pyraustinae*sensu lato* along with various Spilomelinae. This group has been relatively well studied in Europe and North America. In other regions, however, particularly the Oriental Region, this work is far from complete. One of the major contributions to the knowledge of the Pyraustinae of East Asia is a series of papers by Munroe and Mutuura (1968, 1969, 1970, 1971) treating many pyraustine genera of temperate East Asia, a historical milestone in the study of the Pyraustinae of this area. Both authors’ generic concepts, however, were narrow, so that many genera recognized by these authors were united by Tränkner et al. (2009) in a wider concept of the genus *Anania* Hübner.

In recent years, a series of similar yellowish specimens collected from the south of China, all superficially resembling species of *Pseudopagyda* Slamka, 2013, attracted our attention. By examining the genitalia, three described species, *Paligacontractalis* Warren, 1896, *Botysaureolalis* Lederer, 1863, *Botyspraepandalis* Snellen, 1890 and four unknown species were recognized. According to characters of male and female genitalia, they are congeneric, but obviously do not match the genitalic morphology of *Pseudopagyda* or *Paliga* Moore, 1886. Bänziger (1995) placed *Paligacontractalis* and *Botysaureolalis* in genus *Microstega* Meyrick, 1890 along with *Pioneaacutangulata* Swinhoe, 1901 and *Microstegahomoculorum* Bänziger, 1995. Bänziger (1995) also pointed out that *M.homoculorum* and *M.acutangulata* are congeneric, but that *M.contractalis* and *M.aureolalis* probably each belong to a different genus, and that *Pioneapraepandalis* resembles *M.aureolalis* superficially, without giving any details. *Microstega* was synonymized with *Paratalanta* Meyrick, 1890 by Kirpichnikova (1986) and Maes (1994) based on “the characteristic sclerotized hook (spicula-shaped sella) on the valvae of the male genitalia”. This taxonomic decision is commonly accepted, but Zhang et al. (2014) excluded the above five species from *Paratalanta* because they share no generic synapomorphies with *Paratalanta*. Almost at the same time, Slamka (2013) proposed genus *Pseudopagyda* for *M.homoculorum*. Subsequently, *M.acutangulata* was transferred to *Pseudopagyda* (Chen and Zhang 2017). Slamka (2013) also suggested that *Paligacontractalis* and *Botysaureolalis* should belong to *Pseudopagyda*. However, in a revision of *Pseudopagyda* (Chen and Zhang 2017), several putative synapomorphic characters of the genus were summarized, and it was found that *Paligacontractalis* and *Botysaureolalis* are not congeneric with species of *Pseudopagyda* based on genitalia characters.

After comparing these species with taxonomic treatments, faunal surveys, and checklists of Spilomelinae and Pyraustinae (Hampson 1893, 1896, 1898, 1899, Caradja 1925, Shibuya 1928, 1929, Munroe and Mutuura 1968, 1969, 1970, 1971, Munroe 1976a, 1976b, Wang 1980, Inoue 1982, Heppner and Inoue 1992, Munroe 1995, Speidel 1996, Shaffer et al. 1996, Kirpichnikova 1999, 2009, Wang and Speidel 2000, Mathew 2006, Shaffer and Munroe 2007, Bae et al. 2008, Leraut 2012, Slamka 2013, Yamanaka et al. 2013, Scholtens and Solis 2015) and type specimens deposited in the Natural History Museum, London, the Zoological Institute, Academy of Sciences of Russia, St. Petersburg, the Australian National Insect Collection and the National Museum of Natural History Grigore Antipa, Bucharest, Romania, our efforts of placing these species in a suitable genus were unsuccessful. Moreover, they can’t be placed in any African pyraustine genus (Dr Koen VN Maes, pers. comm.). The seven species treated here, currently with no appropriate generic placement, could be easily separated from other pyraustine taxa by several genital traits in both males and females, especially the peculiar uncus, for which the erection of a new genus is considered warranted.

Thus, the aim of this study is to propose a new genus, provide several synapomorphic characters, present an identification key based on external features and genitalia, redescribe three known species, and describe four new ones. A preliminary phylogenetic analysis of the genus and of several potentially related genera, is also proposed based on molecular data.

## Materials and methods

### Molecular material and methods

All species of the genus *Spinosuncus*, two species of the genus *Pseudopagyda*, and four species of other genera of Pyraustinae were included for molecular phylogenetic analysis (Table 1). *Pseudebuleafentoni* Butler, 1881 was chosen as outgroup because it was considered as a basal lineage of the Pyraustinae (Zhang 2003).

**Table 1.** Species sampled for the molecular phylogenetic analysis.

Genus	Species	Voucher number	Locality	GenBank accession number			Reference
				COI	16S	EF-1α	
* Pseudebulea *	* fentoni *	SYSU-LEP0074	Hunan Prov.	MG739570	MG739582	MG739594	Chen et al. 2018
* Paratalanta *	* ussurialis *	SYSU-LEP0158	Hunan Prov.	MK000093	MK000070	MK000116	present study
* Ostrinia *	* furnacalis *	SYSU-LEP0157	Jiangxi Prov.	MK000094	MK000071	MK000117	present study
* Placosaris *	* rubellalis *	SYSU-LEP0087	Jiangxi Prov.	MK000095	MK000072	MK000118	present study
* Thliptoceras *	* sinense *	SYSU-LEP0080	Jiangxi Prov.	MK000096	MK000073	MK000119	present study
* Aglaops *	* youboialis *	SYSU-LEP0068	Jiangxi Prov.	MK000097	MK000074	MK000120	present study
* Pseudopagyda *	* homoculorum *	SYSU-LEP0116	Yunnan Prov.	MK000098	MK000075	MK000121	present study
	* acutangulata *	SYSU-LEP0011	Jiangxi Prov.	MK000099	MK000076	MK000122	present study
	* acutangulata *	SYSU-LEP0126	Jiangxi Prov.	MK000100	MK000077	MK000123	present study
* Spinosuncus *	* aureolalis *	SYSU-LEP0132	Yunnan Prov.	MK000101	MK000078	MK000124	present study
	* aureolalis *	SYSU-LEP0146	Yunnan Prov.	MK000102	MK000079	MK000125	present study
	* quadracutus *	SYSU-LEP0001	Hainan Prov.	MK000103	MK000080	MK000126	present study
	* quadracutus *	SYSU-LEP0002	Hainan Prov.	MK000104	MK000081	MK000127	present study
	* curvisetaceus *	SYSU-LEP0129	Jiangxi Prov.	MK000105	MK000082	MK000128	present study
	* praepandalis *	SYSU-LEP0006	Guizhou Prov.	MK000106	MK000083	MK000129	present study
	* praepandalis *	SYSU-LEP0131	Yunnan Prov.	MK000107	MK000084	MK000130	present study
	* brevacutus *	SYSU-LEP0009	Guizhou Prov.	MK000108	MK000085	MK000131	present study
	* brevacutus *	SYSU-LEP0010	Guizhou Prov.	MK000109	MK000086	MK000132	present study
	* brevacutus *	SYSU-LEP0156	Jiangxi Prov.	MK000110	MK000087	MK000133	present study
	* rectacutus *	SYSU-LEP0134	Guizhou Prov.	MK000111	MK000088	MK000134	present study
	* rectacutus *	SYSU-LEP0155	Guizhou Prov.	MK000112	MK000089	MK000135	present study
	* contractalis *	SYSU-LEP0133	Yunnan Prov.	MK000113	MK000090	MK000136	present study
	* contractalis *	SYSU-LEP0135	Yunnan Prov.	MK000114	MK000091	MK000137	present study
	* contractalis *	SYSU-LEP0153	Yunnan Prov.	MK000115	MK000092	MK000138	present study

Total DNA was extracted from one hindleg and one midleg of 24 specimens using the TIANGEN DNA extraction kit following the manufacturer’s instructions. The nucleotide sequences of two mitochondrial genes, cytochrome c oxidase subunit I (COI) and 16S ribosomal RNA (16S rRNA), and one nuclear gene, elongation factor-1 alpha (EF-1α), were selected for study. Primers used in this study were chosen according to Simon et al. (2006), Wahlberg and Wheat (2008) and Hundsdörfer et al. (2009). PCR cycle conditions were an initial denaturation of 5 min at 95 °C, 30 s at 94 °C, 30 s at 48 °C (COI and 16S rRNA) or 51 °C(EF-1α), and 1 min at 72 °C for 35 cycles, and a final extension at 72 °C for 10 min. All amplifications were confirmed by gel electrophoresis on a 1.5% W/V agarose gel in TAE buffer. PCR products were direct-sequenced at Majorbio Bio-pharm Technology Co., Ltd (Guangzhou), utilizing the same primers used for PCR amplification.

The sequences were aligned using Clustal W (Thompson et al. 1994) under default settings. Gaps were treated as missing data in all analyses. Phylogenetic analyses were conducted using Bayesian inference (BI) method and Maximum likelihood (ML). The BI analysis was run in MrBayes 3.2.6 (Ronquist et al. 2012) with independent parameters for the gene partitions for COI and 16S rRNA under the GTR+G model and for the EF-1α gene partition under the SYM+I+G model as suggested by jModelTest 0.1.1 (Posada 2008). Two independent runs, each with four Markov Chain Monte Carlo (MCMC) simulations, were performed for 3 million generations sampled every 1000^th^ step. The first 25% of the trees were discarded as burn-in, and posterior probabilities (PP) were determined from remaining trees. The ML analysis was executed in RAxML 8.2.10 (Stamatakis 2014) for all gene partitions under the GTR + G model proposed by jModelTest 0.1.1 (Posada 2008) and with 1000 iterations for bootstrap test. The pairwise Kimura 2-Parameter (K2P) distances between species were calculated from the COI gene using MEGA 6 (Tamura et al. 2013).

### Morphological materials and methods

The specimens studied, including the types of the newly described species, are all deposited at the Museum of Biology, Sun Yat-sen University, Guangzhou (**SYSBM**) except those specified as being in the Insect Collection of the College of Life Sciences, Nankai University (**NKU**), the Natural History Museum, London, United Kingdom (**NHMUK**) and the Forest Canopy Ecology Lab, Yunnan (**FCEL**). Slides of genitalic dissections were prepared according to Robinson (1976) and Li and Zheng (1996), with some modifications. Genitalia terms follow Klots (1970), Munroe (1976a), Maes (1995), and Kristensen (2003). Specimen images at different focal levels were made using a Canon EOS 1DX camera (provided with a Canon 100 mm macro lens) in combination with Helicon Remote. Genitalia pictures were taken using a Zeiss Axio Scope.A1 in combination with a Zeiss AxioCam camera and the Axio Vision SE64 program on a Windows PC; source images were then aligned and stacked on Helicon Focus to obtain a fully sharpened composite image. All the pictures were edited using Adobe Photoshop CS5.

## Results

### Phylogenetic relationships

The concatenated dataset of three genes consisted of 1863 nucleotide positions (658 for COI, 434 for 16S rRNA and 771 for EF-1α, respectively). Pairwise distances of the barcode region (COI) are given in Table 2. The genetic distances between the genus *Spinosuncus* (described below) and the other genera range from 9.0% (*Aglaops*) to 17.0% (*Pseudebulea*). Interspecific genetic distances within *Spinosuncus* range from 2.5% (*S.contractalis* to *S.rectacutus*) to 13.8% (*S.aureolalis* to *S.rectacutus*) while intraspecific genetic distances in *Spinosuncus* range from 0% (*S.contractalis*) to 2.7% (*S.aureolalis*).

**Figure 1.**
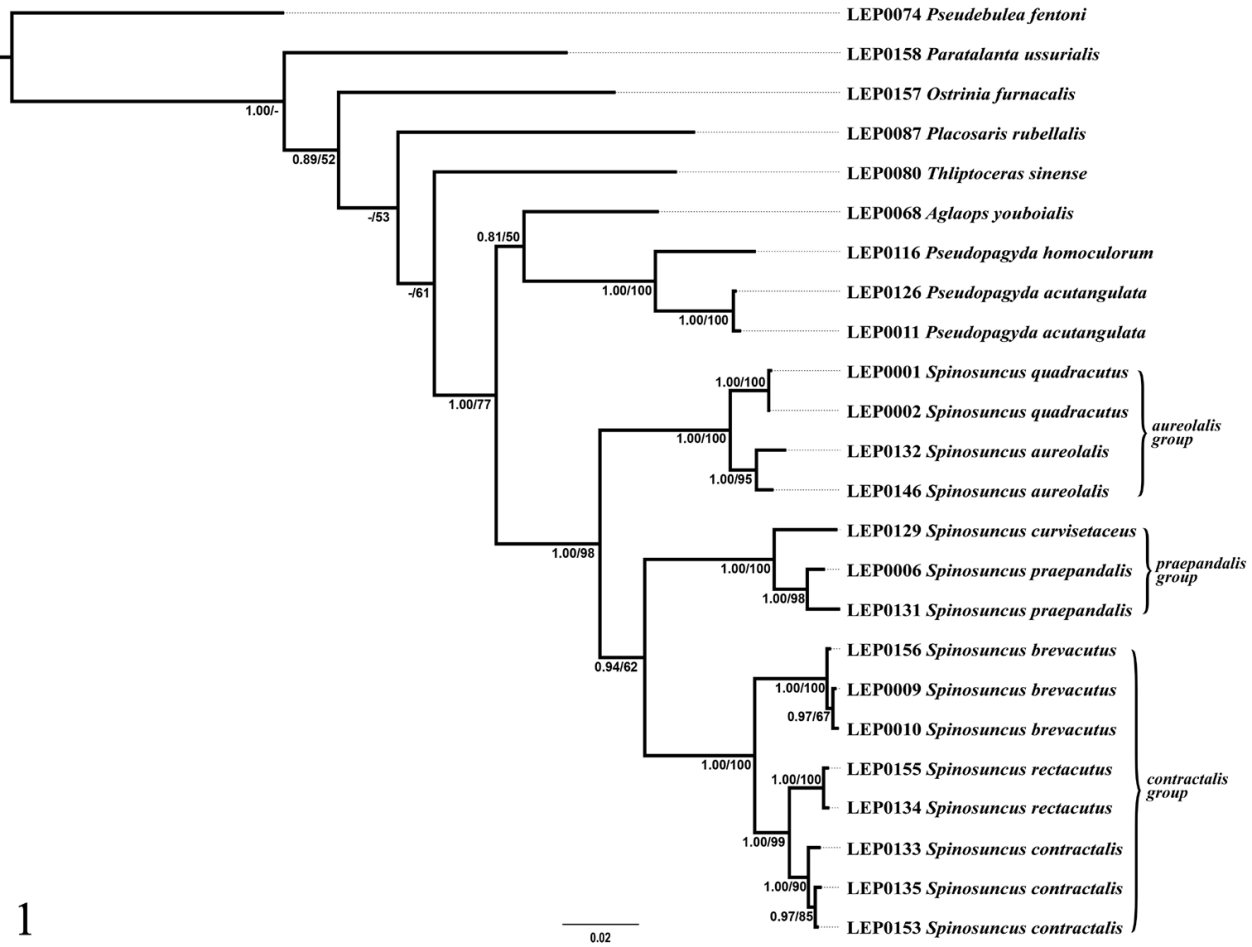
Phylogenetic hypothesis inferred from Bayesian inference. Numbers on branches indicate Bayesian posterior probabilities (values ≥ 0.8 are labelled) and ML bootstrap values probabilities (values ≥ 50% are labelled), respectively.

The BI and ML analyses of the concatenated dataset inferred congruent topologies with only subtle differences in posterior probability and bootstrap values probability (Figure 1). The monophyly of *Spinosuncus* is robustly supported (PP = 1.00, BS = 98). Within *Spinosuncus*, three well-supported clades are identified. The clade *S.aureolalis* + *S.quadracutus*, clade *S.curvisetaceus* + *S.praepandalis* and clade *S.brevacutus* + (*S.rectacutus* + *S.contractalis*) are each recovered with robust supports (PP = 1.00, BS = 100). Clade *Aglaopsyouboialis* + (*Pseudopagydahomoculorum* + *P.acutangulata*) is in a sister position to clade *Spinosuncus* with robust support as well (PP = 1.00, BS = 77). Distances between *Spinosuncus* and *Pseudopagyda* range from 10.2% to 13.3%, and between *Spinosuncus* and *Aglaops* from 9.0% to 12.9%.

**Table 2.** Pairwise distances of the COI barcode region based on Kimura-2-parameter model (intraspecific distances are highlighted in bold).

		1	2	3	4	5	6	7	8	9	10	11	12	13	14	15	16	17	18	19	20	21	22	23
1	**LEP0132 *Spinosuncusaureolalis***																							
2	**LEP0146 *Spinosuncusaureolalis***	**0.027**																						
3	**LEP0001 *Spinosuncusquadracutus***	0.044	0.037																					
4	**LEP0002 *Spinosuncusquadracutus***	0.046	0.035	**0.002**																				
5	**LEP0129 *Spinosuncuscurvisetaceus***	0.111	0.092	0.109	0.107																			
6	**LEP0006 *Spinosuncuspraepandalis***	0.115	0.100	0.107	0.105	0.041																		
7	**LEP0131 *Spinosuncuspraepandalis***	0.117	0.107	0.105	0.107	0.050	**0.024**																	
8	**LEP0009 *Spinosuncusbrevacutus***	0.129	0.104	0.118	0.116	0.083	0.101	0.109																
9	**LEP0010 *Spinosuncusbrevacutus***	0.131	0.106	0.120	0.118	0.085	0.103	0.111	**0.002**															
10	**LEP0156 *Spinosuncusbrevacutus***	0.125	0.104	0.114	0.116	0.083	0.101	0.105	**0.003**	**0.005**														
11	**LEP0134 *Spinosuncusrectacutus***	0.138	0.112	0.120	0.118	0.094	0.100	0.109	0.049	0.047	0.049													
12	**LEP0155 *Spinosuncusrectacutus***	0.138	0.112	0.120	0.118	0.094	0.100	0.109	0.047	0.046	0.047	**0.005**												
13	**LEP0133 *Spinosuncuscontractalis***	0.131	0.106	0.118	0.116	0.089	0.095	0.099	0.049	0.048	0.049	0.027	0.025											
14	**LEP0135 *Spinosuncuscontractalis***	0.127	0.106	0.114	0.112	0.089	0.095	0.095	0.049	0.048	0.049	0.027	0.025	**0.003**										
15	**LEP0153 *Spinosuncuscontractalis***	0.127	0.106	0.114	0.112	0.089	0.095	0.095	0.049	0.048	0.049	0.027	0.025	**0.003**	**0.000**									
16	**LEP0068 *Aglaopsyouboialis***	0.129	0.112	0.118	0.116	0.090	0.100	0.104	0.094	0.096	0.094	0.098	0.098	0.100	0.100	0.100								
17	**LEP0116 *Pseudopagydahomoculorum***	0.131	0.124	0.133	0.130	0.102	0.114	0.114	0.124	0.126	0.124	0.118	0.118	0.120	0.116	0.116	0.085							
18	**LEP0011 *Pseudopagydaacutangulata***	0.129	0.122	0.126	0.126	0.108	0.122	0.120	0.124	0.126	0.122	0.130	0.130	0.122	0.118	0.118	0.092	0.058						
19	**LEP0126 *Pseudopagydaacutangulata***	0.129	0.122	0.126	0.126	0.108	0.122	0.120	0.124	0.126	0.122	0.130	0.130	0.122	0.118	0.118	0.092	0.058	0.000					
20	**LEP0080 *Thliptocerassinen***se	0.149	0.138	0.135	0.133	0.129	0.138	0.134	0.131	0.133	0.131	0.137	0.135	0.131	0.129	0.129	0.096	0.118	0.123	0.123				
21	**LEP0087 *Placosarisrubellal***is	0.133	0.124	0.135	0.137	0.100	0.110	0.119	0.114	0.116	0.110	0.116	0.120	0.116	0.116	0.116	0.087	0.112	0.112	0.112	0.127			
22	**LEP0157 *Ostriniafurnacalis***	0.142	0.124	0.120	0.118	0.126	0.130	0.137	0.116	0.118	0.116	0.133	0.133	0.130	0.130	0.130	0.108	0.114	0.132	0.132	0.120	0.112		
23	**LEP0158 *Paratalantaussurialis***	0.143	0.126	0.143	0.141	0.122	0.133	0.139	0.122	0.124	0.122	0.124	0.128	0.124	0.124	0.124	0.102	0.120	0.112	0.112	0.135	0.106	0.102	
24	**LEP0074 *Pseudebuleafentoni***	0.148	0.135	0.148	0.146	0.159	0.161	0.159	0.161	0.158	0.161	0.170	0.170	0.161	0.158	0.158	0.158	0.133	0.143	0.143	0.161	0.145	0.145	0.157

Since the monophyly of *Spinosuncus* is well-supported and species within the clade are morphologically and genetically distinct from the potential sister groups, a new genus is proposed. The taxonomic details are provided below.

### Taxonomy

#### Spinosuncus gen. n.

Taxon classificationAnimaliaLepidopteraCrambidae

http://zoobank.org/AF399C02-2BDC-48D6-9A57-384D3DD6F5AD

##### Type species.

*Paligacontractalis* Warren, 1896

##### Diagnosis.

Species of *Spinosuncus* can be recognized externally by the yellow to fulvous wing ground colour, the fulvous to brown lines, the distinct subterminal lines usually arched to CuA_2_ then obviously angled or concave near the tornus. Diagnostic characters in the male genitalia are the short and stout, strongly sclerotized uncus distally with two spines or teeth, the lamellate, distally inflated sella set with fin-shaped setae forming editum, the dorsally inflated sacculus with the dorsal margin sclerotized and usually spinulose, the distally broad and usually spinulose phallus, and the spine-like cornuti appear funnel-shaped in the distal end of the vesica. The female genitalia are characterized by the strongly sclerotized lamella postvaginalis always extended dorsolaterally, and the sclerotized transverse band posteriorly in the cup-shaped antrum.

*Spinosuncus* moths are most similar in appearance to *Pseudopagyda* Slamka, 2013. Some species of *Spinosuncus* can be distinguished by the much smaller wingspan (usually less than 24 mm). However, some *Spinosuncus* species have a similar body size to *Pseudopagyda*, but they can still be differentiated by the wavy or dentate lines on the wings dorsally, especially the sinuate (rather than oblique, or slightly curved as in *Pseudopagyda*) anterior part of the postmedial line near the costa. In the male genitalia, the sclerotized uncus, the fin-shaped setae (editum) of the sella, and the inflated sacculus distinguish *Spinosuncus* from *Pseudopagyda*. In the female genitalia, the long and slender ductus bursae is distinct from the extremely short ductus bursae of *Pseudopagyda*.

##### Description.

**Figure 2.**
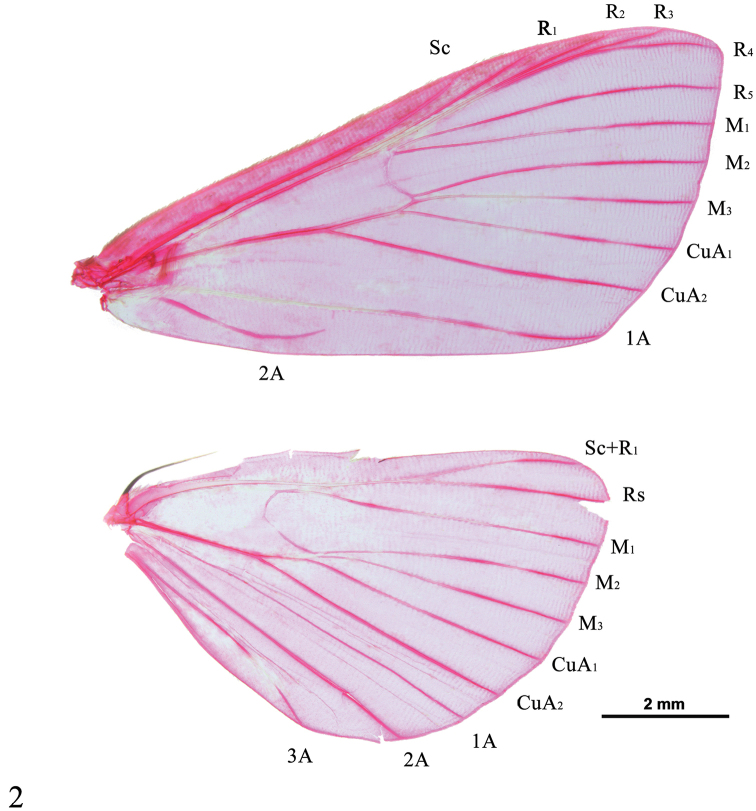
Wing venation of *Spinosuncuspraepandalis*.

*Head*. Frons oblique, yellowish brown, with white lateral bands. Vertex with moderately raised scales projecting between antennae. Labial palpus obliquely upturned, exceeding frons by 2/3 length of head or slightly less, third palpomere porrect, yellowish brown with base contrastingly white. Maxillary palpus small, yellowish brown, tips pale yellow, sometimes mixed with white. Proboscis well developed, with basal scaling white. Antenna pale yellow, with cilia as long as width of corresponding flagellomeres in male. *Thorax*. With appressed scales, yellow. Legs unmodified. Foreleg brown, tibia white with brown cross band medially, tarsus white; midleg pale brown, tibia and tarsus white ventrally; hind leg pale yellow, tinged with white, basal inner spur longer than apical inner spurs. Forewing subtriangular, termen gently arched; retinaculum a tuft of curved bristles from below base of discal cell. Hindwing fan-shaped, costal margin translucent whitish; frenulum simple in male, with two acanthae in female. Wing venation (Figure 2) in forewing with cell about half length of wing; R_1_ free, from 4/5 of anterior margin of cell, R_2_ free but adjacent to stem of R_3_+R_4_ in about basal half, R_3_ and R_4_ stalked to about 2/3, R_4_ to just before apex, R_5_ parallel to stalked R_3_+R_4_ at base then diverging; M_1_ moderately close to R_5_ at base, M_2_ widely separate from M_1_, closing vein concavely curved; M_2_, M_3_ and CuA_1_ from posterior angle of cell, M_3_ closer to M_2_ at base than to CuA_1_, then diverging; CuA_2_ from 3/5 of posterior margin of cell; 1A faintly sinuate to tornus, 2A forming complete loop and distally recurved before joining 1A, sometimes disconnected. Hindwing with cell about 1/3 length of wing; Sc+R_1_ and Rs anastomosing for 1/3 beyond end of discal cell, Rs and M_1_ short-stalked, closing vein concave, angled medially; M_3_ closer to M_2_ at base than to CuA_1_, parallel with M_2_ at base, then diverging; 1A complete but weak, 3A curved. *Abdomen*. Slender, usually yellowish, sometimes dark brown, apical margin of segments usually tinged with white. *Male genitalia.* Uncus short and stout, nearly quadrate, with wide base; usually strongly sclerotized; distal end with two or four sharp spines laterally or distally bifid forming two teeth; glabrous or ventrolaterally set with few setae, or densely setose at base of teeth. Tegumen quadrate. Vinculum U-shaped. Saccus short, near triangular, rounded at apex. Valva tongue-shaped, varying in width, tapering towards apex, set with hair-like setae on inner side; transtilla sub-triangular, meeting in middle, usually with setae on dorsal margin; costa simple, costal sclerotized band narrow to broad, extended to beyond 2/3 of dorsal margin; sacculus broad, expanded except basal part, with dorsal margin strongly sclerotized and often spinose; sella slender to broad, lamellate, distally inflated, set with modified setae (editum), varying from fin-shaped to thick, needle-shaped. Juxta heart-shaped to nearly pentagonal. Phallus with distal part broad and moderately setose, usually spinulose; vesica in distal part with numerous spine-like cornuti appear funnel-shaped, sometimes with several large spicules. *Female genitalia*. Ovipositor lobes flat, densely setose. Sinus vaginalis well developed, membranous, usually with sclerotized, streak-like or hook-like notches anterolaterally (absent in *S.praepandalis* and *S.curvisetaceus*); lamella postvaginalis band-shaped, sclerotized (weakly sclerotized in *S.contractalis*, *S.rectacutus* and *S.brevacutus*), always extended dorsolaterally. Antrum membranous or sclerotized and granulated, cup-shaped, with sclerotized transverse band posteriorly. Ductus seminalis originating from anterior end of colliculum. Ductus bursae with base slightly rotated, as long as or longer than length or diameter of corpus bursae; colliculum ring-shaped, sclerotized. Corpus bursae drop-shaped or globular; accessory bursa present, sometimes with second signum at base; main signum rhomboid.

##### Biology.

All of the Chinese material has been collected during the night at light. Host information is currently unavailable. *Spinosuncusaureolalis* and *S.contractalis* occur sympatrically with species of *Pseudopagyda* in some places. According to Bänziger (1995), they are not lachryphagous.

##### Distribution.

*Spinosuncus* occurs in South China (Figure 28), India, and Thailand.

##### Etymology.

The generic name is a compound word that refers to the uncus distally with spines (“*spinosus*” in Latin). The resultant name is masculine in gender.

#### Spinosuncus contractalis

Taxon classificationAnimaliaLepidopteraCrambidae

(Warren, 1896) comb. n.

**Figures 3–9.**
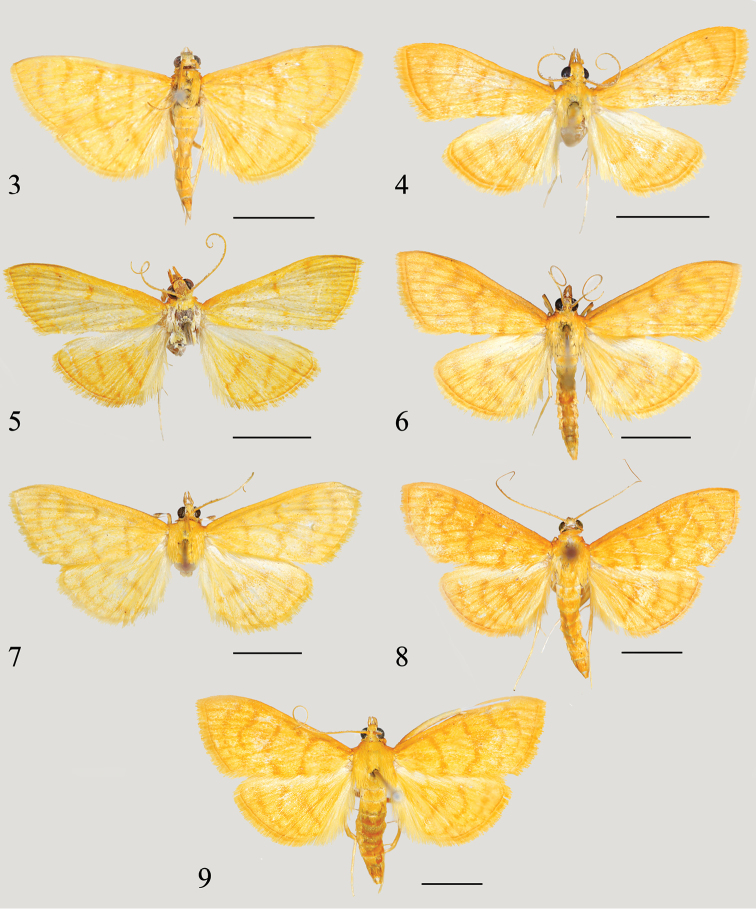
Adults of *Spinosuncus* spp. **3***S.contractalis*, male (Dahaoping, Yunnan) **4***S.rectacutus*, holotype, male (Weng’ang Town, Guizhou) **5***S.brevacutus*, holotype, male (Weng’ang Town, Guizhou) **6***S.praepandalis*, male (Weng’ang Town, Guizhou) **7***S.curvisetaceus*, paratype, male (Tongmu Village, Fujian) **8***S.aureolalis*, male (Bubang, Yunnan) **9***S.quadracutus*, paratype, male (Mt. Limu, Hainan). Scale bars: 5.0 mm.

**Figures 10–12.**
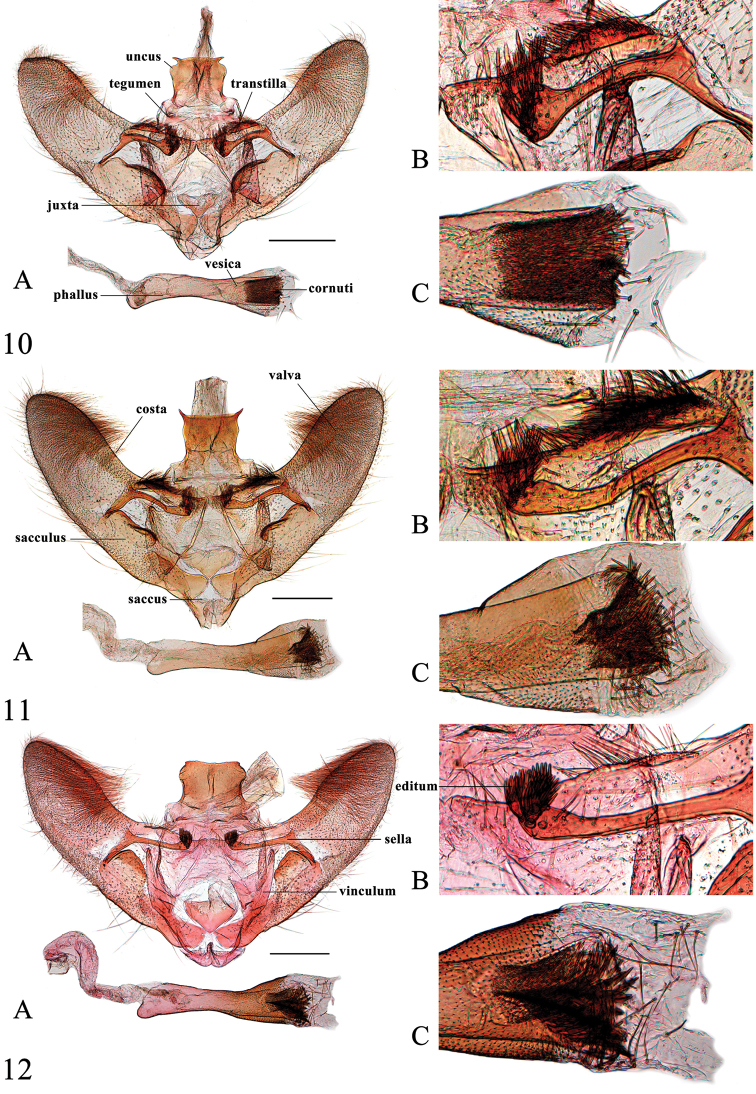
Male genitalia of *Spinosuncus* spp. **10***S.contractalis*, Hainan (genitalia slide no. SYSU0017) **11***S.rectacutus*, Guangxi (genitalia slide no. SYSU0044) **12***S.brevacutus*, Guizhou (genitalia slide no. SYSU0910). **A**: Whole genitalia. **B**: Base of valva dorsally. **C**: Apex of phallus. Scale bars: 0.5 mm.

Figs 3 , 10 , 19 , 28

Paliga contractalis Warren, 1896, 18(6): 123.
Microstega contractalis (Warren) Bänziger, 1995: 270.

##### Material examined.

**Type material.** Lectotype, 1♂; Khasis, Warren Type, Pyralidae Brit. Slide No. 8677 (NHMUK), designated by Bänziger (1995).

##### Other material examined.

**CHINA, Hainan**: 2♂, 1♀, Bawangling, Changjiang, 19.12N, 109.08E, alt. 161 m, 22.VII.2014, leg. Cong Peixin, Hu Sha and Liu Linjie, genitalia slide no. ZDD12049 (♂) (NKU); 1♀, Bawangling, 11.VI.2010, leg. Kang Li, genitalia slide no. SYSU0185; 1♂, 1♀, Jianfengling, 5.VI.2010, leg. Kang Li, genitalia slide no. SYSU0174 (♂); 1♀, Jianfengling, 18.75N, 108.85E, alt. 969 m, 12.IX.2013, leg. Xie Weicai, genitalia slide no. SYSU0067; 1♂, Bangxi Reserve, 19.37N, 109.10E, alt. 97 m, 2.IX.2013, leg. Chen Xiaohua, genitalia slide no. SYSU0017; 1♂, Nankai Town, Baisha, 19.05N, 109.40E, alt. 294 m, 19.V.2013, leg. Li Jinwei, genitalia slide no. SYSU0065; 1♂, Sanya Village, Fanjia, 19.25N, 109.65E, alt. 302 m, 27.X.2013, leg. Chen Kai and Chen Xiaohua, genitalia slide no. SYSU0040; 1♀, Mt. Diaoluoshan, alt. 500 m, 24.V.2014, leg. Xu Dan and Xu Lijun, genitalia slide no. SYSU0914; 1♀, Wuzhishan Natural Reserve, 18.88N, 109.65E, alt. 742 m, 21.V.2015, leg. Cong Xinpei, Guan Wei and Hu Sha (NKU); **Yunnan**: 3♂, Bawan, Baoshan, alt. 1040 m, 9.VIII.2007, leg. Zhang Dandan, genitalia slide no. SYSU0019; 5♂, 1♀, Baihualing, Baoshan, alt. 1520 m, 11,13.VII.2007, leg. Zhang Dandan, genitalia slides no. CXH12155 (♂), SYSU0039 (♂), SYSU0047 (♂), SYSU0073 (♀); 2♂, Baihualing, Mt. Gaoligongshan, Baoshan City, 25.30N, 98.80E, alt. 1473 m, 29.VII.2013, leg. Liu Shurong, Teng Kaijian and Wang Yuqi (NKU); 1♂, Baihualing, Mt. Gaoligongshan, Baoshan City, 25.30N, 98.80E, alt. 1473 m, 7.VIII.2014, leg. Liu Shurong, Rong Hua and Teng Kaijian (NKU); 1♂, Dahaoping, alt. 2020 m, 6.VIII.2007, leg. Zhang Dandan; 1♂, Jingpozhai, Nabang, Yingjiang, 24.71N, 97.39E, alt. 231 m, 3.VIII.2013, leg. Liu Shurong, Teng Kaijian and Wang Yuqi (NKU); 2♂, 1♀, 55 km site, Xishuangbanna Natural Reserve, 23.V.2015, leg. Zhang Zhenguo, genitalia slide no. ZDD12053 (♂, molecular voucher no. SYSU-LEP0153) (NKU); 1♂, Yexiang Valley, Xishuangbanna, 22.17N, 100.87E, alt. 762 m, 18.VII.2014, leg. Guan Wei, Liu Shurong and Wang Xiuchun (NKU); 2♂, 1♀, Yexiang Valley, Xishuangbanna, 22.17N, 100.87E, alt. 762 m, 10–12.VII.2015, leg. Bai Xia and Teng Kaijian, genitalia slide no. ZDD12048 (♂, molecular voucher no. SYSU-LEP0135) (NKU); 1♂, Guanping, Mengyang, alt. 1200 m, 20.VIII.2005, leg. Ren Yingdang, genitalia slide no. CYP12056 (NKU); 1♂, Nanla River, Bubang, Mengla, 21.59N, 101.58E, alt. 652 m, 15.VII.2013, leg. Liu Shurong, Teng Kaijian and Wang Yuqi (NKU); 1♀, Yaoqu Town, Xishuangbanna, alt. 780 m, 26.V.2015, leg. Tao Manfei, genitalia slide no. SYSU0913, molecular voucher no. SYSU-LEP0133; **Tibet**: 1♀, Medog, alt. 1103 m, 8.VII.2013, leg. Li Jinwei, genitalia slide no. SYSU0915.

##### Diagnosis.

Within the genus, *S.contractalis* resembles *S.rectacutus* and *S.brevacutus* in the relatively small wingspan, the almost indistinguishable wing pattern, the glabrous uncus, a row of dense setae on the transtilla dorsally, the two sclerotized notches anterolaterally on the sinus vaginalis and the short ductus bursae (approximately as long as the length of the corpus bursae). However, it can be differentiated from *S.rectacutus* by the somewhat more sinuate postmedial line of the forewing near costa, in the male genitalia by the shorter, excurved spines of the uncus and the acinaciform, densely spinous extension of the sacculus distally. In the female genitalia, it is characterized by the curved sclerotized notches anterolaterally on the sinus vaginalis. The differences between *S.contractalis* and *S.brevacutus* are given in the diagnosis of the latter species.

##### Redescription.

*Head*. As for the genus. *Thorax*. Yellow. Legs as described for the genus. Wingspan 18–22 mm. Wings yellow, lines fulvous. Forewing broadly triangular with moderately arched termen; antemedial line weakly sinuate from about 1/4 of costa to 2/5 of posterior margin; orbicular stigma small, sometimes faint; reniform stigma a fulvous, slightly curved streak; posterior angle of cell outwardly followed by a fulvous mark; postmedial line from 3/5 of costa slightly sinuate to beyond basal half of CuA_1_, bent inwardly to 1/3 of CuA_2_, then to 2/3 of posterior margin; subterminal line from distal end of R_2_, arched to about 4/5 of CuA_2_, then concave to 4/5 of posterior margin; fringe yellowish brown. Hindwing with costa and posterior margin translucent whitish; posterior angle of cell outwardly followed by a fulvous mark; postmedial line straight from basal half of M_1_ to distal third of CuA_2_, bent inwardly to basal third of CuA_2_, then straight to near end of 2A; subterminal line from distal third of R_S_, arched, tapering to CuA_2_, then concave to distal end of 1A; fringe as in forewing. *Abdomen*. Yellow dorsally, apical margin of segments tinged with white. *Male genitalia* (Figure 10). Uncus with lateral margin strongly bulging near distal end, with a sharply widened base; without setae; with two outwardly curved, pointed spines, weakly dentate between the spines. Valva of medium width, slightly narrowing towards apex, length approximately 2× its maximal width; transtilla dorsally strongly sclerotized and set with dense setae; costal sclerotized band narrow, slightly expanded to 2/3 of dorsal margin; sacculus with distal half expanded, forming acinaciform sclerotized process, dorsally set with dense spines; sella long and slender, rod-like, distal end strongly inflated, set with several narrow, fin-shaped setae forming editum, each seta with apex evenly divided into several filaments. Juxta heart-shaped, deeply divided distally. Phallus with distal 1/3 expanded and spinulose; vesica in distal part with numerous spine-like cornuti appear funnel-shaped (Figure 10C). *Female genitalia* (Figure 19). Posterior apophysis with small expansion at basal third; anterior apophysis with small expansion beyond basal half. Sinus vaginalis with two curved, sclerotized notches anterolaterally; lamella postvaginalis weakly sclerotized medially, most strongly sclerotized dorsolaterally. Antrum membranous. Ductus bursae moderately broad, nearly as long as length of corpus bursae; colliculum narrow medially. Corpus bursae approximatively drop-shaped; accessory bursa arising from posterior 1/3 of corpus bursae, with small, densely spinulose second signum beside its base; rhombic signum with two opposing angles bearing well developed carinae and closely separated medially, the other two angles bearing dense spines.

##### Distribution.

(Figure 28). China (Hainan, Yunnan, Tibet), India, Thailand.

#### Spinosuncus rectacutus sp. n.

Taxon classificationAnimaliaLepidopteraCrambidae

http://zoobank.org/B77B43A4-3F97-4157-ACD1-BD646B953C4D

Figs 4 , 11 , 20 , 28

##### Material examined.

**Type material.** Holotype ♂ (Fig. 4); **CHINA, Guizhou**: Weng’ang Town, Maolan Reserve, Libo, 25.25N, 107.90E, alt. 814 m, 25.VII.2015, leg. Chen Kai, genitalia slide no. SYSU0060. Paratypes: **Hubei**: 1♂, Pingbaying, Xianfeng, alt. 1280 m, 21.VII.1999, leg. Li Houhun et al., genitalia slide no. ZDD12055 (NKU); 3♂, Mahe Town, Xianfeng, alt. 400 m, 24–26.VII.1999, leg. Li Houhun et al., genitalia slide no. ZDD12056 (NKU); 1♂, Maobaqu, Lichuan, alt. 700 m, 29.VII.1999, leg. Li Houhun et al., genitalia slide no. ZDD12057 (NKU); **Guangxi**: 1♂, Nanchao, Yachang Forest Farm, Leye, alt. 1160 m, 26.VII.2004, leg. Xu Jiasheng (NKU); 1♂, Huaping, Yachang Forest Farm, Leye, alt. 910 m, 28.VII.2004, leg. Xu Jiasheng, genitalia slide no. CYP12058 (NKU); 15♂, 2♀, Nonggang, Longzhou, 22.47N, 106.96E, alt. 271 m, 19.IV.2012, leg. Li Jinwei, genitalia slides no. SYSU0036 (♀), 0188 (♀), 0009, 0014, 0024, 0041, 0042, 0043, 0044, 0054, 0055; 8♂, 2♀, Nonggang, Longzhou, alt. 188 m, 25,27,28,31.VII.2011, leg. He Guiqing, genitalia slides no. SYSU0189 (♂), 0194 (♀), 0979 (♀); 1♂, Nonggang Reserve, 21.VIII.2011, leg. Yang Lijun, genitalia slide no. SYSU0053; 1♂, Nonggang Reserve, 21.VIII.2011, leg. Cheng Muchun, genitalia slide no. CXH12165; 1♂, Nonggang, Longzhou, 20.VIII.2011, leg. Cheng Muchun; 1♂, Nonggang, Longzhou, alt. 280 m, 29.VII.2012, leg. Yang Xiaofei (NKU); 1♂, Sanlian, Longzhou, alt. 180 m, 1.VIII.2011, leg. He Guiqing; 1♂, Tongling Valley, 23.02N, 106.65E, alt. 535 m, 22.VII.2013, leg. Chen Xiaohua, genitalia slide no. SYSU0259; 1♂, Longrui Reserve, 18.VIII.2011, leg. Li Jinwei, genitalia slide no. SYSU0021; 1♂, Longrui Reserve, 19.VIII.2011, leg. Zhang Dandan; 2♂, Bangliang, Jingxi, 1,5.VIII.2010, leg. Huang Jianhua, genitalia slide no. ZDD12047 (NKU); **Guizhou**: 2♂, Xian’nv’dong, Dashahe, Daozhen, alt. 600 m, 28–29.V.2004, leg. Hao Shulian, genitalia slide no. CYP12057 (NKU); 1♂, Baishao Village, Qinggangtang, Suiyang, alt. 800 m, 11.VIII.2010, leg. Du Xicui, genitalia slide no. CYP12063 (NKU); 1♂, Baishao, Kuankuoshui, alt. 800 m, 10.VIII.2010, leg. Du Xicui, genitalia slide no. SYSU0187; 1♂, Mt. Leigongshan, 26.35N, 108.15E, alt. 1198 m, leg. Chen Xiaohua, genitalia slide no. SYSU0057, molecular voucher no. SYSU-LEP0155; 1♂, Dongdai, Shuizu Town, Limingguan, Libo, alt. 720 m, 19.VII.2015, leg. Li Jia’en and Yang Meiqing, genitalia slide no. ZDD12050, molecular voucher no. SYSU-LEP0134 (NKU); 1♀, Weng’ang Town, Maolan Reserve, Libo, 25.25N, 107.90E, alt. 814 m, 25.VII.2015, leg. Chen Kai, genitalia slide no. SYSU0072; **Chongqing**: 2♂, 1♀, Xiaonanhai, Qianjiang, alt. 370 m, 21.VII.2012, Xu Lijun and Zhang Jun, genitalia slides no. SYSU0186 (♂), 0193 (♀).

##### Diagnosis.

*Spinosuncusrectacutus* resembles *S.contractalis* and *S.brevacutus*, for which details are provided in the diagnosis of *S.contractalis*. It can be best distinguished from *S.brevacutus* by the dorsally densely setose transtilla (moderately setose in *S.brevacutus*), and the saddle-shaped sacculus with sclerotized margin densely set with a row of spinules. The distal spines of the uncus are straight and longer than those of *S.brevacutus*, and the lateral margin near the distal end of the uncus is less bulging. In the female genitalia, the length of the colliculum is approximately 1.5× as long as its minimal width and the notches on the sinus vaginalis are strongly sclerotized whereas in *S.brevacutus*, the length of the colliculum is approximately as long as its minimal width and the notches on the sinus vaginalis are weakly sclerotized.

##### Description.

*Head*. As for the genus. *Thorax*. Yellow. Legs as described for the genus. Wingspan 18–22.5 mm. Wing pattern as in *S.contractalis*. *Abdomen*. Yellow dorsally, apical margin of segments tinged with white. *Male genitalia* (Figure 11). Uncus with lateral margin slightly bulging near distal end, with base sharply widened; setae absent; distal two corners with straight, pointed spines, outer margin between spines dentate. Valva of medium width, length approximately 2.3× its maximal width; transtilla with dorsal margin strongly sclerotized and set with dense setae; costal sclerotized band wide, slightly expanded to 2/3 of dorsal margin; distal half of sacculus expanded to a saddle-shaped structure, with sclerotized margin, basal half of margin slightly twisted, set with dense spines; sella long and slender, rod-like, distal end slightly inflated, set with several narrow, fin-shaped setae forming editum, each seta with apex evenly divided into several filaments. Juxta heart-shaped. Phallus as in *S.contractalis*. *Female genitalia* (Figure 20). Posterior apophysis with small expansion at basal third; anterior apophysis with small expansion beyond basal half. Sinus vaginalis with two straight, sclerotized notches anterolaterally; lamella postvaginalis weakly sclerotized medially, most strongly sclerotized dorsolaterally. Antrum membranous. Ductus bursae slender, nearly as long as length of corpus bursae; colliculum narrow medially. Corpus bursae drop-shaped, slightly spinulose; accessory bursa arising from posterior 1/3 of corpus bursae, with small, densely spinulose second signum beside its base; rhombic signum with carinae almost connected.

##### Etymology.

The specific name is derived from the Latin *recti*- for straight and *acutus*, pointed, referring to the straight, pointed spines of the uncus.

##### Distribution.

(Figure 28). China (Hubei, Guangxi, Guizhou, Chongqing).

#### Spinosuncus brevacutus sp. n.

Taxon classificationAnimaliaLepidopteraCrambidae

http://zoobank.org/D2E5D324-0CEB-46A5-B31E-4B520E704B72

Figs 5 , 12 , 21 , 28

##### Material examined.

**Type material.** Holotype ♂ (Fig. 5); **CHINA, Guizhou**: Weng’ang Town, Maolan Reserve, Libo, 25.25N, 107.90E, alt. 814 m, 25.VII.2015, leg. Chen Kai, genitalia slide no. SYSU0056, molecular voucher no. SYSU-LEP0009. Paratypes: **Jiangxi**: 1♂, Main Peak, Mt. Jinggangshan, 28.IV.2011, leg. Liu Ping and Mei Yan, genitalia slide no. CXH12192; 1♂, Main Peak, Mt. Jinggangshan, 30.VI.2011, leg. Yang Lijun, genitalia slide no. CXH12161; 1♂, Main Peak, Mt. Jinggangshan, 1.IX.2011, leg. Cheng Muchun, genitalia slide no. SYSU0064; 1♂, 1♀ (Abdomen lost), Reservoir of Mt. Jinggangshan, 19.IX.2010, leg. Tong Bo, Zhang Dandan and Zhao Shuang; 1♂, 1♀, Mt. Guanggushan, Wuzhifeng Town, Shangyou, 25.92N, 114.05E, alt. 846 m, 22.VI.2015, leg. Chen Kai, genitalia slides no. SYSU0015 (♂), 0062 (♀, molecular voucher no. SYSU-LEP0156); 1♂, Mt. Jiulianshan, Longnan, 24.54N, 114.46E, alt. 625 m, 28.IV.2012, leg. Li Jinwei, genitalia slide no. SYSU0049; **Hunan**: 1♀, Visitors’ center, Taoyuandong, 26.47N, 114.04E, alt. 870 m, 20.V.2014, leg. Chen Xiaohua, genitalia slide no. SYSU0063; **Guizhou**: 1♂, Maolan Reserve, 1.IX.2011, leg. Li Jinwei, genitalia slide no. CXH12162; 4♂, Maolan Reserve, 25.13N, 107.87E, alt. 797 m, 12.VII.2013, leg. Chen Xiaohua, genitalia slides no. SYSU0020, 0023, 0074, 0910; 1♂, 2♀, Weng’ang Town, Maolan Reserve, Libo, 25.25N, 107.90E, alt. 814 m, 25.VII.2015, leg. Chen Kai, genitalia slides no. SYSU0046 (♂), 0071 (♀, molecular voucher no. SYSU-LEP0010), 0978 (♀).

##### Diagnosis.

*Spinosuncusbrevacutus* is similar to *S.contractalis* and *S.rectacutus*. Differences with *S.rectacutus* are given in the diagnosis of *S.rectacutus*. It can be distinguished from *S.contractalis* by the minute and weakly outwardly curved spines of the apical uncus, the concave margin between those spines, the moderately setose transtilla and the semicircular sacculus distally with sclerotized, sparsely toothed margin in the male genitalia, by the straight, weakly sclerotized notches of the sinus vaginalis (curved, strongly sclerotized in *S.contractalis*) and the relatively broad ductus bursae in the female genitalia.

##### Description.

*Head*. As for the genus. *Thorax*. Yellow. Legs as described for the genus. Wingspan 19–24 mm. Wing pattern as in *S.contractalis*. *Abdomen*. Yellow dorsally, apical margin of segments tinged with white. *Male genitalia* (Figure 12). Uncus with the lateral margin strongly bulging near distal end, with base sharply widened; setae absent; distal two corners slightly extended, forming minute spines. Valva of medium width, length approximately 2.5× its maximal width; transtilla with dorsal margin slightly sclerotized, set with few setae; costal sclerotized band rather wide, slightly expanded to 2/3 of dorsal margin; distal half of sacculus expanded, semicircular, with strongly sclerotized margin, sometimes set with few tiny teeth, distal third of margin twisted; sella long and slender, rod-like, distal end slightly inflated and upcurved, set with several narrow, fin-shaped setae forming editum, each seta with apex evenly divided into several filaments. Juxta heart-shaped, distal half divided. Phallus as in *S.contractalis*. *Female genitalia* (Figure 21). Posterior apophysis with small expansion at basal third; anterior apophysis with small expansion beyond basal half. Sinus vaginalis with two straight, weakly sclerotized notches anterolaterally; lamella postvaginalis weakly sclerotized medially, most strongly sclerotized dorsolaterally. Antrum membranous. Ductus bursae moderately broad, as long as length of corpus bursae; colliculum somewhat constricted medially. Corpus bursae drop-shaped; accessory bursa arising from posterior 1/3 of corpus bursae, with small, weakly spinulose second signum beside its base; rhombic signum with two opposing angles bearing weak, narrow carinae almost connected medially, the other two angles set with spines.

##### Etymology.

The specific name is derived from the Latin *brevi*-, short, and *acutus* for pointed, referring to the short, pointed spines of the uncus.

##### Distribution.

(Figure 28). China (Jiangxi, Hunan, Guizhou).

#### Spinosuncus praepandalis

Taxon classificationAnimaliaLepidopteraCrambidae

(Snellen, 1890) comb. n.

**Figures 13–14.**
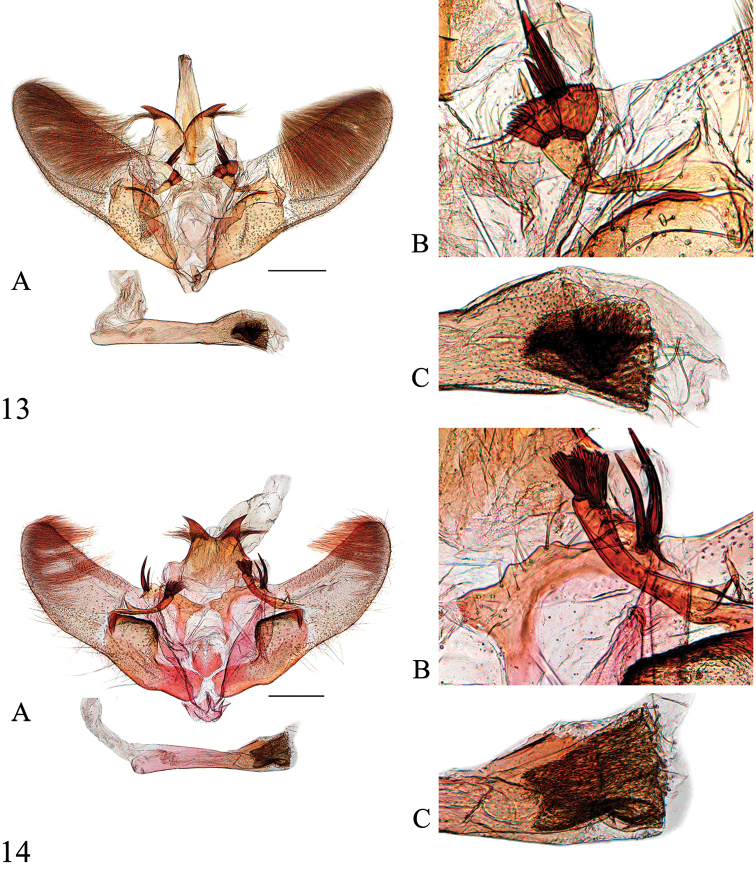
Male genitalia of *Spinosuncus* spp. **13***S.praepandalis*, Guizhou (genitalia slide no. SYSU0038) **14***S.curvisetaceus*, Fujian (genitalia slide no. ZDD12051). **A**: Whole genitalia. **B**: Base of valva dorsally. **C**: Apex of phallus. Scale bars: 0.5 mm.

Figs 6 , 13 , 22 , 28

Botys praepandalis Snellen, 1890: 573–574.

##### Material examined.

**Type material.** Lectotype, 1♀; Sikkim, O. Miller., [18]89, collection of H. J. Elwes, Pyralidae Brit. Slide no. 9711 (NHMUK).

##### Other material examined.

**CHINA, Hubei**: 1♂, 1♀, Shayuan, Hefeng, alt. 1260 m, 15,17.VII.1999, leg. Li Houhun, genitalia slides no. ZDD02388 (♂), 02389 (♀) (NKU); Hunan: 1♀, Jiangping, Mt. Hupingshan, Shimen County, alt. 480 m, 6.V.2002, leg. Yu Haili (NKU); **Sichuan**: 1♂, Wannian Temple, Mt. E’meishan, 29.59N, 103.38E, alt. 830 m, 14.VII.2014, leg. Guan Wei, Liu Shurong and Wang Xiuchun (NKU); **Chongqing**: 1♂, Dawopu, Mt. Simianshan, 28.58N, 106.35E, alt. 1059 m, 12.VII.2016, leg. Chen Kai; 1♂, Tiantangba, Mt. Simianshan, 28.64N, 106.35E, alt. 921 m, 13.VII.2016, leg. Chen Kai; 1♂, Mt. Jinfoshan, alt. 1700 m, 13.VII.2010, leg. Du Xicui and Shi Shengwen, genitalia slide no. SYSU0191; 1♀, Wuli Town, Qianjiang, alt. 870 m, 23.VII.2012, leg. Xu Lijun and Zhang Jun, genitalia slide no. SYSU0196; **Guizhou**: 1♂, Heiwan, Jiangkou, alt. 600 m, 28.VII.2001, leg. Li Houhun and Wang Xinpu, genitalia slide no. ZDD02061 (NKU); 2♂, Huguo Temple, Mt. Fanjingshan, alt. 1390 m, 28.V.2002, leg. Wang Xinpu, genitalia slide no. CYP12041 (NKU); 3♂, Weng’ang Town, Maolan Reserve, Libo, 25.25N, 107.90E, alt. 814 m, 25.VII.2015, leg. Chen Kai, genitalia slide no. SYSU0038, molecular voucher no. SYSU-LEP0006; 1♂, Weng’ang Town, Libo, alt. 1345 m, 18.VII.2015, leg. Wan Jiping; **Yunnan**: 4♂, 2♀, Mt. Jizushan, Binchuan, 25.93N, 100.38E, alt. 1831 m, 29.VI.2012, leg. Li Jinwei, genitalia slides no. CXH12156 (♂), SYSU0045 (♂), 0076 (♀), 0980 (♀); 5♂, 1♀, Mt. Gaoligongshan, Baoshan, 24.82N, 98.78E, alt. 1700 m, 22.V.2016, leg. Duan Yongjiang, genitalia slides no. SYSU0190 (♂), 0195 (♀, molecular voucher no. SYSU-LEP0131); 2♀, Dahaoping, Tengchong, alt. 2020 m, 6.VIII.2007, leg. Zhang Dandan, genitalia slides no. SYSU0078, 0183; 1♂, Pianma Village, Lushui, Nujiang, alt. 1889 m, 16.VIII.2015, leg. Wei Xueli; 1♀, Malipo County, alt. 1098 m, 4.VI.2015, leg. Tao Manfei, genitalia slide no. SYSU0911; **Tibet**: 1♂, 1♀, Hanmi, Medog, alt. 2380 m, 9.VIII.2003, leg. Wang Xinpu and Xue Huaijun, genitalia slide no. CYP12062 (♂) (NKU); 1♂, Shangzayü, Nyingchi, alt. 1936 m, 16.VIII.2015, leg. Xu Dan. **INDIA**: 1♂, India, Sikkim, Elwes, collection of H. J. Elwes, Pyralidae Brit. Slide no. 8674 (NHMUK); 1 ♂, Sikkim, O. Miller., [18]89, collection of H. J. Elwes (NHMUK).

##### Diagnosis.

*Spinosuncuspraepandalis* has a larger wingspan (24–30 mm) than in the species described above. It has a wingspan similar to that of *S.aureolalis*, but can be differentiated by the dentate lines and the thickened anterior part of the postmedial line of the forewing near the costa. In the male genitalia, it is distinguished by the distally bifid uncus, forming two sclerotized, large outwardly curved teeth with a hairy basal margin (as in *S.curvisetaceus*), the two to three straight, thick needle-shaped setae dorsally set on each side of the transtilla and the semicircular sacculus distally with the margin sclerotized and with a small process distally. In the female genitalia, it is distinguished by the sinus vaginalis without sclerotized, streak-like or hook-like notches (as in *S.curvisetaceus*) and the long and slender ductus bursae, which is more than twice as long as the diameter of the corpus bursae, differs from that of the species described above (the ductus bursae is almost as long as the length of the corpus bursae). The differences between *S.praepandalis* and *S.curvisetaceus* are given in the diagnosis of the latter species.

##### Redescription.

*Head*. As for the genus. *Thorax*. Yellow. Legs as described for the genus. Wingspan 24–30 mm. Wing pattern as in *S.contractalis*, apart from: wings yellowish brown; lines brown and wavy; postmedial line of forewing thickened near costa, strongly sinuate to half of CuA_1_; postmedial line of hindwing curved to distal third of CuA_2_. *Abdomen*. Yellowish to brown, apical margin of segments tinged with white. *Male genitalia* (Figure 13). Uncus tapering towards apex; distal 3/4 bifid, forming two outwardly curved, strongly sclerotized teeth, medially set with dense setae, arranged in a curved line. Valva of medium width, ventral margin beyond sacculus slightly concave, length approximately 2.3× its maximal width; transtilla extended ventrally into a projection, each lobe set with two to three straight, thick needle-shaped setae at dorsal base (one seta occasionally falls off), with one much bigger than other(s); costal band moderately wide, slightly expanded to 2/3 of dorsal margin; distal half of sacculus expanded, semicircular, with dorsal margin sclerotized, apically with small, triangular process; sella long and slender, rod-like, upcurved (bent in Figs 13A–B), distal end slightly inflated, set with a few broad, fin-shaped setae forming editum, each seta with apex evenly divided into several filaments. Juxta pentagonal, weakly bifid distally. Phallus with distal 1/4 slightly expanded and spinulose; vesica in distal part with numerous spine-like cornuti appear funnel-shaped (Figure 13C). *Female genitalia* (Figure 22). Posterior apophysis with distinct hook-like expansion at basal 2/5. Sinus vaginalis without sclerotized, streak-like or hook-like notches; lamella postvaginalis band-shaped, well developed, extended to cover entire eighth segment ventrally. Antrum membranous, with a narrow sclerotized transverse band posteriorly. Ductus bursae long and slender, more than three times as long as diameter of corpus bursae; colliculum almost evenly wide. Corpus bursae small, globular; accessory bursa arising from posterior 1/3 of corpus bursae; rhombic signum with well developed, moderately separated carinae, other two angles bearing spines medially, the anterior angle smaller than the posterior angle; second signum absent.

##### Distribution.

(Figure 28). China (Hubei, Hunan, Sichuan, Chongqing, Guizhou, Yunnan, Tibet), India.

#### Spinosuncus curvisetaceus sp. n.

Taxon classificationAnimaliaLepidopteraCrambidae

http://zoobank.org/5E9A3861-D420-43A5-9B85-343125D46FCB

Figs 7 , 14 , 23 , 28

##### Material examined.

Holotype ♂; **CHINA, Jiangxi**: Mt. Sanqingshan, Jinsha County, Shangrao, alt. 380–390 m, 20.IV.2007, leg. Bai Haiyan and Du Xicui, genitalia slide no. ZDD12058 (NKU). Paratypes: **Fujian**: 1♂, Tongmu Village, Mt. Wuyishan, 3.V.2014, leg. Yang Xiaofei, genitalia slide no. ZDD12051 (NKU); **Jiangxi**: 1♂, Shiguling Power Plant, Mt. Sanqingshan, Jinsha County, Shangrao, alt. 410–420 m, 15.IV.2007, leg. Bai Haiyan and Du Xicui, genitalia slide no. CYP12066 (NKU); 6♂, Mt. Sanqingshan, Jinsha County, Shangrao, alt. 380–390 m, 19, 20.IV.2007, leg. Bai Haiyan and Du Xicui, genitalia slides no. CYP12060, 12074, ZDD12026 (NKU); 1♂, Shixi Town, Fengxin, 28.44N, 114.54E, alt. 506 m, 22.IX.2012, leg. Yang Lijun, genitalia slide no. CXH12167; 1♀, Nanfengmian Reserve, Qianmo Village, Suichuan, 26.28N, 114.06E, alt. 816 m, 19.VI.2015, leg. Chen Kai, genitalia slide no. SYSU0061, molecular voucher no. SYSU-LEP0129; **Guangxi**: 1♂, Jiuniutang, Mt. Mao’ershan, alt. 550 m, 20.IV.2002, leg. Hao Shulian and Xue Huaijun, genitalia slide no. ZDD02245 (NKU); 1♂, Huawang Villa, Jinxiu, alt. 550 m, 13.IV.2002, leg. Hao Shulian and Xue Huaijun, genitalia slide no. ZDD02241 (NKU).

##### Diagnosis.

*Spinosuncuscurvisetaceus* resembles *S.praepandalis* in wing pattern. The wingspan of *S.curvisetaceus* is usually smaller than that of *S.praepandalis*, *S.aureolalis* and *S.quadracutus*, but larger than in *S.contractalis*, *S.rectacutus* and *S.brevacutus*. The ground colour of the wings is paler than that of *S.praepandalis*. In the male genitalia, it can be differentiated from *S.praepandalis* by the straight mediobasal margin of the distal teeth of the uncus (curved in *S.praepandalis*), the curved setae on the transtilla dorsally (straight in *S.praepandalis*) and the expanded, rectangular distal half of sacculus, with sclerotized and densely spinulose dorsal margin. In the female genitalia, the anterior apophysis is thicker than that of *S.praepandalis*. It can be distinguished from other *Spinosuncus* species (except *S.praepandalis*) by the distally strongly bifid uncus, forming two sclerotized, large excurved teeth bearing hair-like setae basally, two thick needle-shaped setae on the transtilla dorsally and the absence of sclerotized, streak-like or hook-like notches anterolaterally on the sinus vaginalis.

##### Description.

*Head*. As for the genus. *Thorax*. Yellowish brown. Legs as described for the genus. Wingspan 24–26 mm. Wing pattern as in *S.praepandalis*, ground colour paler than that of *S.praepandalis*. *Abdomen*. Yellowish to brown, apical margin of segments tinged with white. *Male genitalia* (Figure 14). Uncus sharply tapering towards apex; distal half bifid, forming two slightly outwardly curved and sclerotized teeth, basally set with dense setae, arranged in a curved line. Valva of medium width, ventral margin beyond sacculus slightly concave, length approximately 2.1× its maximal width; transtilla extended ventrally into long and curved projection, set with two thick and curved, needle-shaped setae at base dorsally; costal sclerotized band moderately wide, slightly expanded to 3/4 of dorsal margin; distal half of sacculus expanded, rectangular, with dorsal margin strongly sclerotized and densely spinulose, distally twisted; sella long and slender, rod-like, upcurved, distally set with few broad, fin-shaped setae, each seta with apex evenly divided into several filaments. Juxta shield-shaped, distal half divided medially. Phallus as in *S.praepandalis*. *Female genitalia* (Figure 23). Posterior apophysis with hook-like expansion at basal 2/5. Sinus vaginalis without sclerotized, streak-like or hook-like notches; lamella postvaginalis band-shaped, well developed, extended to cover entire eighth segment ventrally. Antrum membranous. Ductus bursae long and slender, more than two times as long as length of corpus bursae; colliculum narrower at anterior end. Corpus bursae small, ovoid; accessory bursa arising from posterior 1/3 of corpus bursae; rhombic signum as in *S.praepandalis*; second signum absent.

##### Etymology.

The specific name is derived from the Latin *curv*- (curved) and *setaceus* (setaceous), referring to the curved setae set at the dorsal base of the transtilla.

##### Distribution.

(Figure 28). China (Fujian, Jiangxi, Guangxi).

#### Spinosuncus aureolalis

Taxon classificationAnimaliaLepidopteraCrambidae

(Lederer, 1863) comb. n.

**Figures 15–16.**
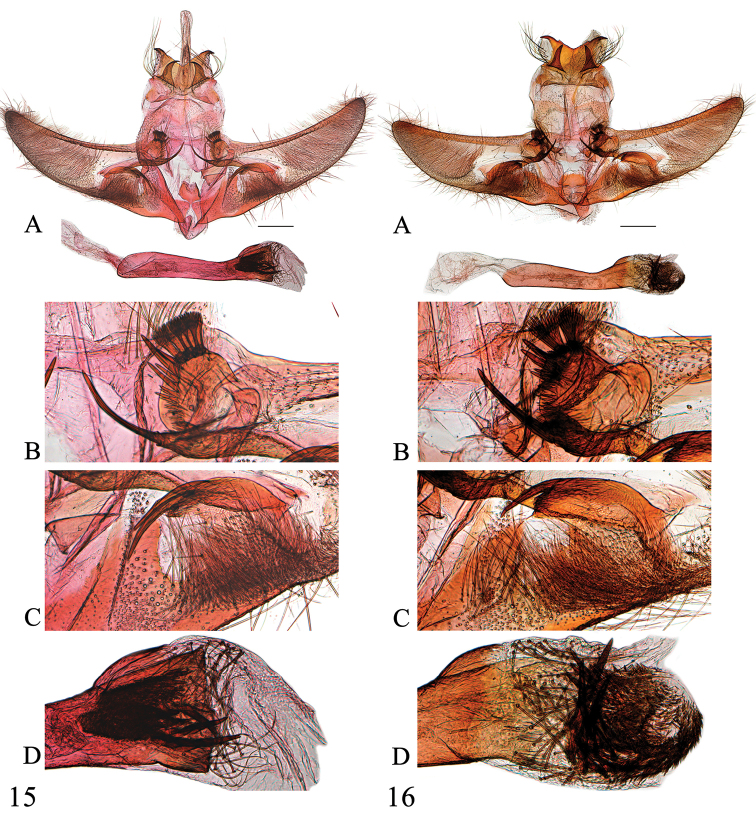
Male genitalia of *Spinosuncusaureolalis*. **15** Yunnan (genitalia slide no. ZDD12054) **16** Yunnan (genitalia slide no. SYSU0173). **A**: Whole genitalia. **B**: Base of valva dorsally. **C**: Projections of sacculus. D: Apex of phallus. Scale bars: 0.5 mm.

Figs 8 , 15–16 , 24–25 , 28

Botys aureolalis Lederer, 1863: 473.
Pyralis ochrealis Moore, 1877: 614.
Microstega aureolalis (Lederer): Bänziger, 1995: 270.

##### Material examined.

**Type material.** Lectotype of *Pyralisochrealis*: 1♂; Sikkim, Moore Coll. 94-106, Pyralidae Brit. Slide No. 8678 (NHMUK), designated by Bänziger (1995).

##### Other material examined.

**CHINA, Guangxi**: 2♂, Nonggang, Longzhou, alt. 188 m, 26.VII.2011, leg. He Guiqing, genitalia slide no. SYSU0909; **Yunnan**: 1♀, Baihualing, Baoshan, alt. 1251 m, 13.VIII.2007, leg. Zhang Dandan, genitalia slide no. SYSU0075; 2♂, Baihualing, Baoshan, alt. 1520 m, 11,13.VIII.2007, leg. Zhang Dandan, genitalia slides no. SYSU0050, 0066; 1♂, 1♀, Mengla, alt. 800 m, 6,8.VII.2012, leg. Kitching and Ashton, genitalia slide no. FCEL0002 (♀) (FCEL); 2♂, 1♀, Bubang, Xishuangbanna, 21.60N, 101.59E, alt. 656 m, 23.VII.2014, leg. Guan Wei, Liu Shurong, Teng Kaijian and Wang xiuchun, genitalia slide no. ZDD12052 (♀, molecular voucher no. SYSU-LEP0146), ZDD12054 (♂) (NKU); 1♂, Nabang, Yingjiang County, 24.75N, 97.56E, alt. 239 m, 27.V.2016, leg. Duan Yongjiang, genitalia slide no. SYSU0958, molecular voucher no. SYSU-LEP0132; 1♂, Pianma Village, Lushui, Nujiang, alt. 1889 m, 16.VIII.2015, leg. Wei Xueli, genitalia slide no. SYSU0959; 1♂, Daxichang, Malipo County, alt. 1465 m, 7.VI.2015, leg. Tao Manfei, genitalia slide no. SYSU0173.

##### Diagnosis.

*Spinosuncusaureolalis* has a large wingspan (more than 26 mm). The ground colour of the wings is the darkest within the genus. Though *S.aureolalis* has a similar wingspan as *S.praepandalis*, it can be distinguished by the sinuate but not thickened anterior part of the postmedial line of the forewing near costa and the smooth, not dentate wing lines. In the male genitalia, it is characterized by the uncus distally with two large spines, the cheliform sacculus projections, and the fin- and needle-shaped setae forming editum on the sella distally (as in *S.quadracutus*). In the female genitalia, the two large, hook-like notches anterolaterally on the sinus vaginalis and the laterally broad, granulated antrum (as in *S.quadracutus*) are diagnostic. The appearance of *S.aureolalis* is most similar to that of *S.quadracutus*, both having the same wing pattern. The differences between these two species are given in the diagnosis of *S.quadracutus*.

##### Redescription.

**Figures 17–18.**
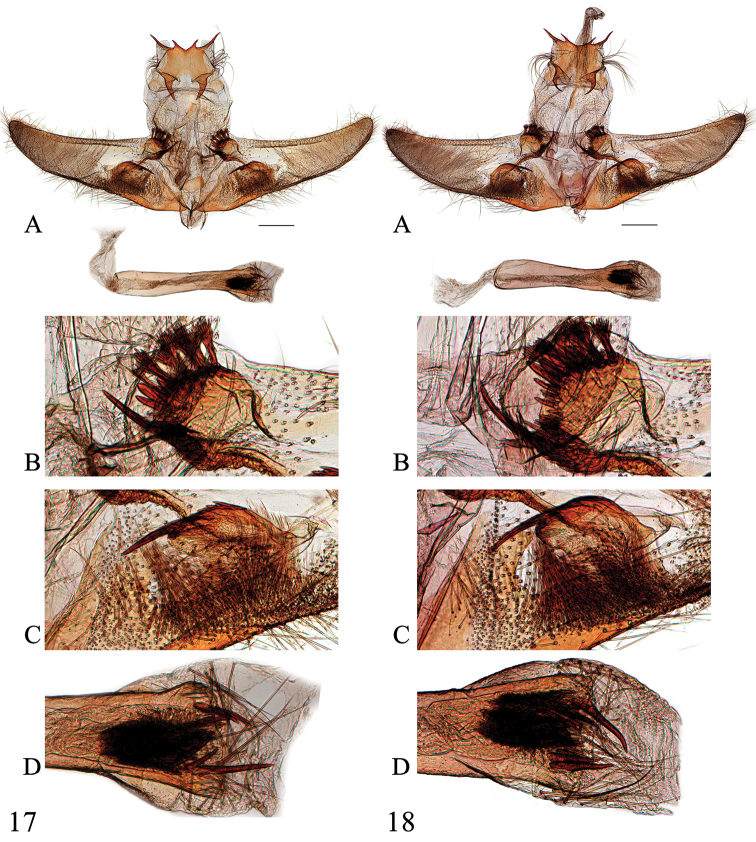
Male genitalia of *Spinosuncusquadracutus*. **17** Fujian (genitalia slide no. SYSU0034) **18** Hainan (genitalia slide no. SYSU0048). **A**: Whole genitalia. **B**: Base of valva dorsally. **C**: Projections of sacculus. **D**: Apex of phallus. Scale bars: 0.5 mm.

*Head*. As for the genus. *Thorax*. Yellow. Legs as described for the genus. Wingspan 26–32 mm. Wings yellow, with fulvous tinge, lines fulvous to yellowish brown, venation somewhat darker than the ground colour, making wings impressively reticulated. Wing pattern as in *S.contractalis*, apart from: postmedial line of forewing more sinuate, of hindwing more curve. *Abdomen*. Fulvous dorsally, apical margin of segments tinged with white. *Male genitalia* (Figs 15, 16). Uncus gradually tapering from base to middle; laterally membranous and set with several setae ventrally, other areas strongly sclerotized; distal 1/3 divided into two sharp teeth, thick, straight or slightly curved (weakly folded in Figs 15A, 16A), between two teeth usually two small and short spines (Figure 16A), sometimes invisible (Figure 15A) (longish, distinct in *S.quadracutus*, Figs 17A, 18A); with two caniniform teeth medioventrally. Valva narrow, length approximately 2.7× its maximal width; transtilla extended ventrally into long and narrow projection, dorsal margin with sparse setae; costal sclerotized band rather narrow, extended to near distal end of valva; sacculus with median caniniform projection and distal cheliform projection, distal half set with dense setae ventrally, distal projection with dorsal margin strongly sclerotized, set with dense and flat-lying spines (except distal half, Figs 15C, 16C) and two moderately downcurved spines pointing towards juxta (sometimes the longer one absent, Figure 16C); sella short and broad, distally inflated, set with modified setae forming editum, varying from fin-shaped to thick needle-shaped, ventral margin upcurved, thickened and sclerotized, distally spinose, ended in long, curved spine. Juxta shield-shaped, pentagonal, distal margin sometimes slightly indented medially. Phallus with distal 1/4 slightly expanded, vesica distally with numerous spinules and several large spicules arranged into funnel-shaped bunch of cornuti (Figs 15D, 16D, rotated in Figure 16D). *Female genitalia* (Figs 24, 25). Anterior apophysis sclerotized, slightly sinuate at distal third; posterior apophysis oblong, slender, strongly sclerotized. Sinus vaginalis with two large, thick, hook-like notches anterolaterally; lamella postvaginalis sclerotized, band-shaped, extended dorsolaterally to about 1/4 width of sinus vaginalis. Antrum granulated and broad. Ductus bursae long and wide, about two times as long as diameter of corpus bursae; colliculum well-developed, with anterior end narrower. Corpus bursae globular; accessory bursa arising from posterior end of corpus bursae; rhombic signum with carinae weak and widely separated, other two angles bearing dense spines; second signum absent.

##### Distribution.

(Figure 28). China (Guangxi, Yunnan), India (Sikkim), Thailand (Chiang Mai).

#### Spinosuncus quadracutus sp. n.

Taxon classificationAnimaliaLepidopteraCrambidae

http://zoobank.org/181BC4CF-DC36-4D53-9084-4AC91688D188

Figs 9 , 17–18 , 26–27 , 28

##### Material examined.

**Type material.** Holotype ♂; **CHINA, Hainan**: Mt. Limushan, 19.16N, 109.73E, alt. 662 m, 20.V.2013, leg. Li Jinwei, genitalia slide no. SYSU0048, molecular voucher no. SYSU-LEP0002. Paratypes: **CHINA, Fujian**: 1♂, Guadun, Mt. Wuyishan, 27.74N, 117.64E, alt. 1220 m, 17.V.2012, leg. Li Jinwei, genitalia slide no. SYSU0034; **Hainan**: 2♂, Mt. Limushan, 19.16N, 109.73E, alt. 662 m, 20.V.2013, leg. Li Jinwei, genitalia slide no. SYSU0032; 1♂, 1♀, Jianling Reserve, 18.87N, 110.27E, alt. 143 m, 8.IX.2013, leg. Chen Xiaohua, genitalia slides no. SYSU0029 (♂), SYSU0035 (♀, molecular voucher no. SYSU-LEP0001); 1♀, Mt. Diaoluoshan, 18.65N, 109.93E, alt. 98 m, 3.XI.2013, leg. Chen Kai and Chen Xiaohua, genitalia slide no. SYSU0912; 1♀, Nankai Town, Baisha, 19.05N, 109.24E, alt. 294 m, 19.V.2013, leg. Li Jinwei, genitalia slide no. SYSU0077.

##### Diagnosis.

This species is indistinguishable from *S.aureolalis* in wing pattern. In the male genitalia, it can be distinguished from *S.aureolalis* by the uncus with four prominent pointed spines distally (the median two small and indistinct in *S.aureolalis*), the blunt distal projection of sacculus (pointed in *S.aureolalis*) always set with one long spine pointing towards juxta (often with two long spines in *S.aureolalis*, Figure 15C) and the more spinulose and with arched dorsal margin distal projection (smooth, less arched in *S.aureolalis*, Figs 15C, 16C). In the female genitalia, it can be differentiated from *S.aureolalis* by the more closely set dorsolateral extensions of lamella postvaginalis and relatively larger and more closely set hook-like notches of the sinus vaginalis anterolaterally (Figs 26B, 27B).

##### Description.

*Head*. Frons brown, vertex with moderately raised scales projecting between antennae, labial palpus brown, white at base ventrally. Maxillary palpus brown, with apex pale yellow. *Thorax*. Yellow. Legs as described for the genus. Wingspan 26–30 mm. Wing pattern as in *S.aureolalis*. *Abdomen*. Fulvous dorsally, apical margin of segments tinged with white. *Male genitalia* (Figs 17, 18). Uncus tapering from base to middle; laterally membranous and set with several setae ventrally; otherwise strongly sclerotized; with two caniniform teeth medioventrally; distally with four sharp and slender spines, the lateral two longer, about two times as long as the median two. Valva narrow, as in *S.aureolalis*; transtilla extended ventrally into a long and narrow projection, dorsal margin with sparse setae; costal sclerotized band rather narrow, extended to near distal end of valva; sacculus with central caniniform projection and distal cheliform projection, distal half set with dense setae ventrally, distal projection strongly sclerotized, set with dense and slightly raised spines and one moderately downcurved spine pointing towards juxta; sella short and broad, distally inflated, set with modified setae forming editum, varying form fin-shaped to thick needle-shaped, ventral margin upcurved, thickened and sclerotized, distally spinose, ending in long, curved spine. Juxta shield-shaped, pentagonal, distal margin slightly bifid. Phallus as in *S.aureolalis*. *Female genitalia* (Figs 26, 27). Anterior apophysis sclerotized, slightly sinuate in distal third; posterior apophysis oblong, slender, and strongly sclerotized. Sinus vaginalis with two large, thick, hook-like notches anterolaterally; lamella postvaginalis sclerotized, band-shaped, extended dorsolaterally to approximately 1/3 width of sinus vaginalis. Antrum granulated and broad. Ductus bursae long and moderately wide, about two times as long as diameter of corpus bursae; colliculum well-developed, with anterior end narrower. Corpus bursae globular; accessory bursa arising from posterior end of corpus bursae; rhombic signum with carinae well-developed and connected (Figure 26A) or weak and wide separated (Figure 27A), other two angles densely bearing spines, sometimes smooth medially (Figure 27A); second signum absent.

**Figures 19–21.**
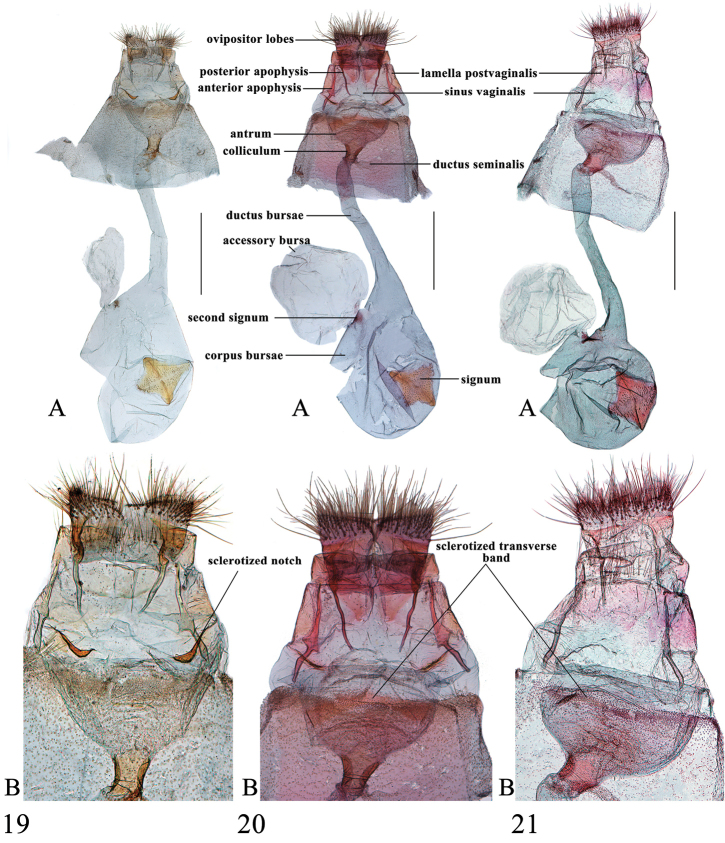
Female genitalia of *Spinosuncus* spp. **19***S.contractalis*, Hainan (genitalia slide no. SYSU0185) **20***S.rectacutus*, Guangxi (genitalia slide no. SYSU0979) **21***S.brevacutus*, Guizhou (genitalia slide no. SYSU0978). A–B: Ventral views. B: Posterad of colliculum. Scale bars: 1.0 mm.

**Figures 22–23.**
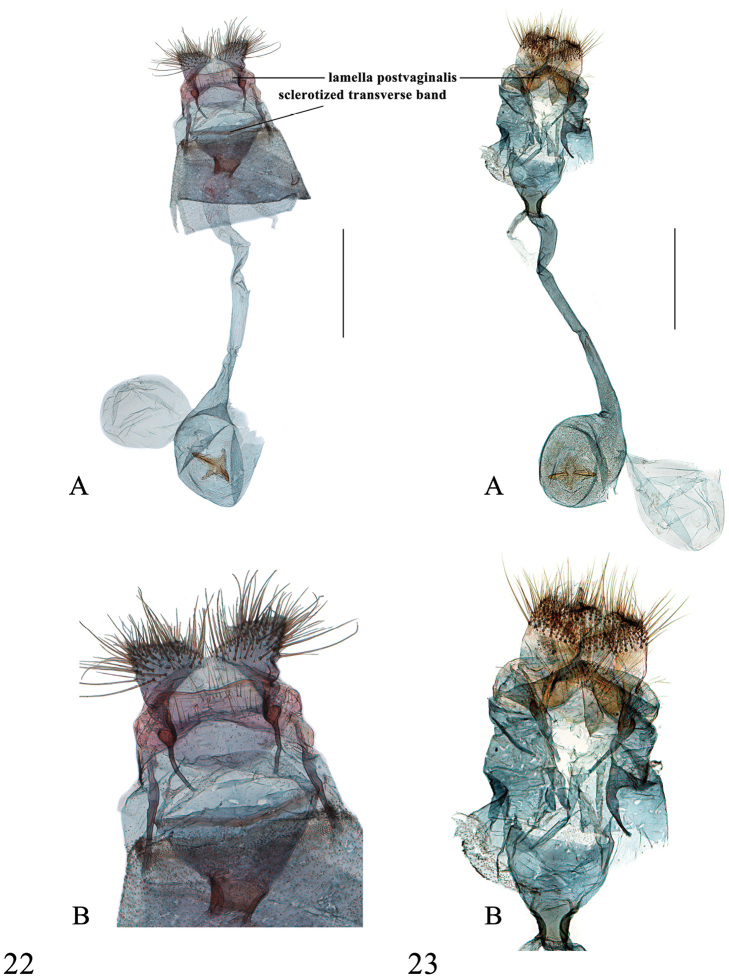
Female genitalia of *Spinosuncus* spp. **22***S.praepandalis*, Yunnan (genitalia slide no. SYSU0980) **23***S.curvisetaceus*, Jiangxi (genitalia slide no. SYSU0061). **A–B**: Ventral views. **B**: Posterad of colliculum. Scale bars: 1.0 mm.

**Figures 24–25.**
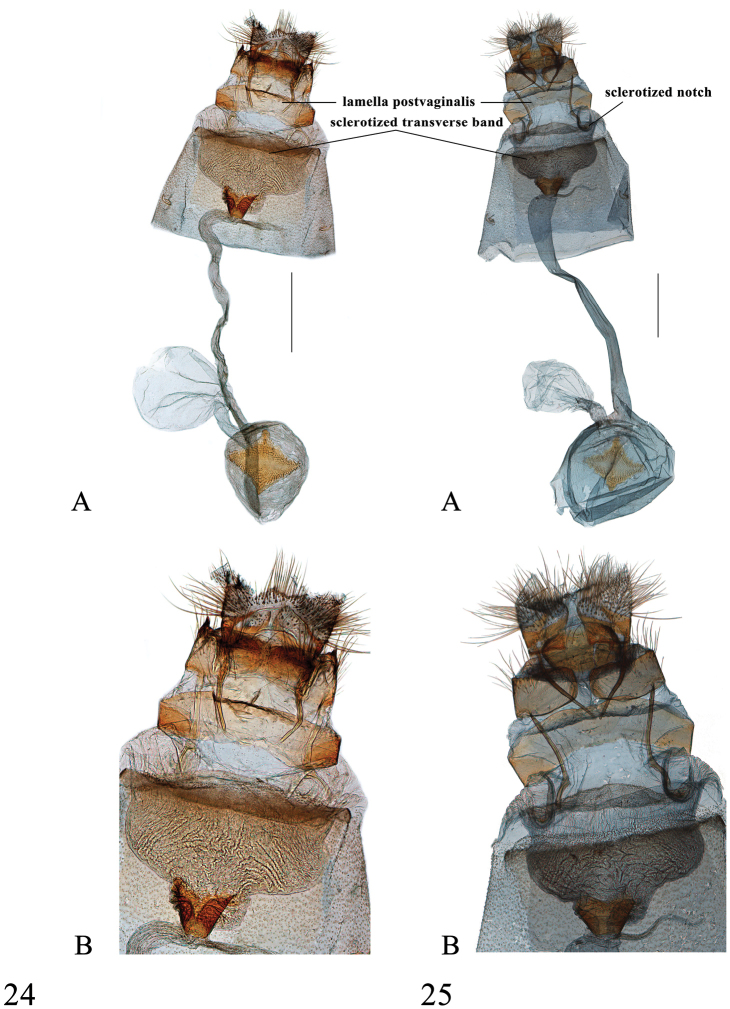
Female genitalia of *Spinosuncusaureolalis*. **24** Yunnan (genitalia slide no. ZDD12052) **25** Yunnan (genitalia slide no. FCEL0002) **A–B**: Ventral views. **B**: Posterad of colliculum. Scale bars: 1.0 mm.

**Figures 26–27.**
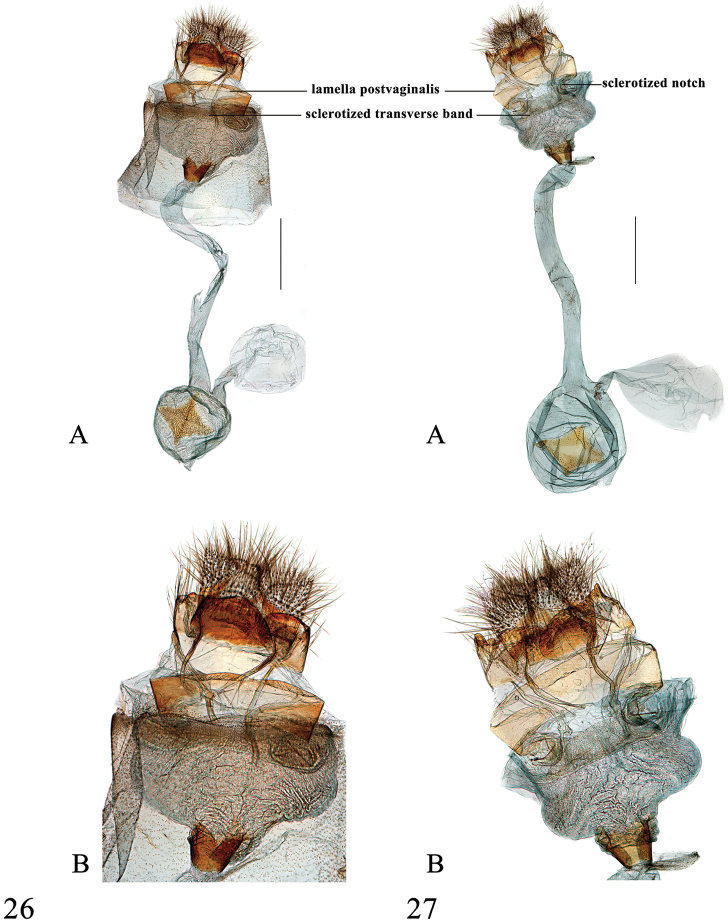
Female genitalia of *Spinosuncusquadracutus*. **26** Hainan (genitalia slide no. SYSU0912) **27** Hainan (genitalia slide no. SYSU0035). A–B: Ventral views. B: Posterad of colliculum. Scale bars: 1.0 mm.

##### Etymology.

The specific name is derived from the Latin *quadri*- (four) and *acutus* (pointed), referring to the distal uncus with four pointed spines.

##### Distribution.

**Figure 28.**
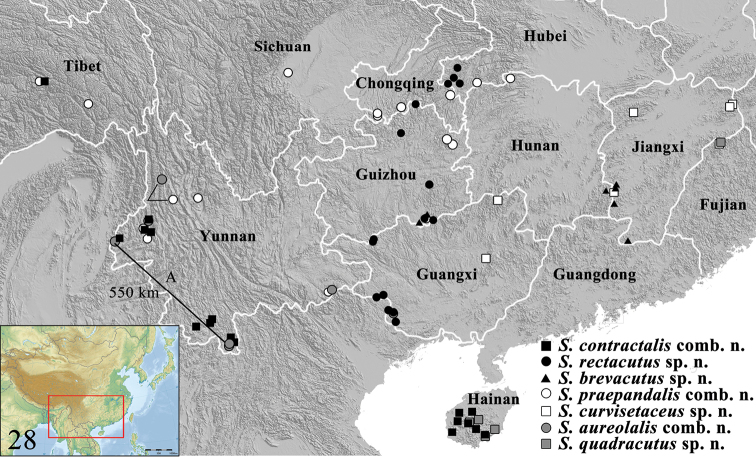
Distribution of *Spinosuncus* species in China. **A** Distance between Bubang and Nabang.

(Figure 28). China (Fujian, Hainan)

### Key to species of *Spinosuncus*

**Table:** 

1	Wingspan large, usually more than 24 mm. Uncus with setae. Ductus bursae at least twice as long as length or diameter of corpus bursae	**2**
–	Wingspan small, usually less than 24 mm. Uncus without setae. Ductus bursae approximately as long as length or diameter of corpus bursae	**5**
2	Forewing with postmedial line thickened near costa. Uncus without teeth ventrally; transtilla with two to three thick needle-shaped setae; sella distally only with fin-shaped setae. Antrum membranous; sinus vaginalis without notch	**3**
–	Forewing with postmedial line not thickened near costa. Uncus with two caniniform teeth ventrally; transtilla with few normal setae; sella distally with fin-shaped and thick needle-shaped setae. Antrum granulated; sinus vaginalis with two large, hook-like notches	**4**
3	Wings with lines somewhat dentate; ground color dark yellow (Fig. 6). Transtilla with two to three straight setae, the longer one about twice as long as the shorter one (Fig. 13B); distal half of sacculus expanded, semicircular, with margin sparsely set with teeth (Fig. 13A)	***S.praepandalis* (Snellen, 1890), comb. n.**
–	Wings with lines not dentate, background color pale yellow (Fig. 7). Transtilla with two curved setae, the longer one less than twice as long as the shorter one (Fig. 14B); distal half of sacculus expanded, rectangular, with margin densely set with teeth (Fig. 14A)	***S.curvisetaceus* sp. n.**
4	Uncus distally with two thick teeth, with two very minute spines, often indistinct (Figs 15A, 16A); sacculus with distal projection relatively pointed, often bearing two long spines pointing towards juxta (Figs 15C, 16C)	***S.aureolalis* (Lederer, 1863), comb. n.**
–	Uncus distally with four slender spines, the lateral two about two times as long as the median two (Figs 17A, 18A); sacculus with distal projection blunt, bearing one long spine pointing towards juxta (Figs 17C, 18C)	***S.quadracutus* sp. n.**
5	Uncus distally blunt, with two minute spines (Fig. 12A); transtilla dorsally with a row of sparse setae (Fig. 12B)	***S.brevacutus* sp. n.**
–	Uncus distally with two distinct spines laterally; transtilla dorsally densely setose	**6**
6	Distal spines of uncus excurved, lateral margins below spines strongly bulging; distal half of sacculus with acinaciform process (Fig. 10A). Notches of sinus vaginalis curved (Fig. 19B)	***S.contractalis* (Warren, 1896), comb. n.**
–	Distal spines of uncus straight, lateral margins below spines slightly bulging; distal half of sacculus without acinaciform process (Fig. 11A). Notches of sinus vaginalis straight (Fig. 20B)	***S.rectacutus* sp. n.**

## Discussion

The results of the molecular analysis robustly support the monophyly of *Spinosuncus*. The monophyly of the genus is further supported morphologically by the following potential synapomorphies: the sclerotized uncus distally with two spines or teeth, the lamellate, distally inflated sella with fin-shaped setae forming editum, the dorsally expanded sacculus with the dorsal margin sclerotized, the distally expanded, spinulose phallus, the funnel-shaped bunch of cornuti, the sclerotized lamella postvaginalis always extended dorsolaterally and the sclerotized transverse band at the posterior end of the antrum.

According to the tree topology (Figure 1), *Paratalanta* is not so closely related to *Spinosuncus*, and as already discussed by Zhang et al. (2014), species of *Spinosuncus* share no synapomorphy with *Paratalanta*. The relationship between *Pseudopagyda* + *Aglaops* and *Spinosuncus* is well supported (PP = 1.00, BS = 77), but the clade *Pseudopagyda* + *Aglaops* is only weakly supported (PP = 0.81, BS = 50). Morphologically, *Aglaops* species are similar to *Pseudopagyda* in the campanulate uncus and the distally inflated sella (which is also similar in *Spinosuncus*), but other genitalia traits differ. The wing pattern of *Aglaops* species lacks a subterminal line, whereas *Pseudopagyda* and *Spinosuncus* species have such a line. *Pseudopagyda* species resemble *Spinosuncus* species in the wing pattern. In the male genitalia, the distally inflated sella is similar to that of *Spinosuncus*; the needle-shaped setae forming editum and the presence of several large spines in the phallus distally are also found in *S.aureolalis* and *S.quadracutus*; the heart-shaped juxta is similar in *S.contractalis*, *S.rectacutus*, *S.brevacutus*, *S.praepandalis* and *S.curvisetaceus*. Based on the molecular phylogenetic results and morphological characters, *Pseudopagyda* could be the most closely related genus to *Spinosuncus*.

Other genera included in the molecular analysis, represented by *Ostriniafurnacalis* (Guenée, 1854), *Placosarisrubellalis* (Caradja, 1925), *Thliptocerassinense* (Caradja, 1925) and *Aglaopsyouboialis* (Munroe & Mutuura, 1968), all lack a forewing subterminal line. *Placosarisrubellalis* and *Thliptocerassinense* have a rod-like sella similar to that of *Spinosuncus* in the male genitalia, but the editum are different. *Ostriniafurnacalis* has a weakly sclerotized uncus, distally divided into three small, laterally setose processes, which is somewhat similar in some *Spinosuncus* species. However, other traits of the male genitalia of *O.furnacalis* are quite different from those of *Spinosuncus* species. At present, it is impossible to confirm the generic position of *Spinosuncus* within the subfamily since only few genera of Pyraustinae were included in this study.

Taxonomically, *Spinosuncus* can be divided into three species groups: the *contractalis* group, the *praepandalis* group and the *aureolalis* group. The monophyly of these three species groups is well supported by the phylogenetic analysis (Figure 1). The *aureolalis* group, comprising *S.aureolalis* and *S.quadracutus*, is well characterized by the laterally setose uncus, distally with two or four spines, ventrally with two large teeth; the lamellate, distally inflated sella with fin- and needle-shaped setae forming editum; the cheliform sacculus with a long spine pointing towards juxta; and the two thick, hook-like notches anterolaterally on the sinus vaginalis. The *contractalis* group comprises *S.contractalis*, *S.rectacutus* and *S.brevacutus*. This species group is well defined by several characters: the glabrous, sclerotized uncus distally with two spines; a row of setae on the transtilla dorsally; and two streak-like sclerotized notches anterolaterally on the sinus vaginalis. Within the *contractalis* group, *S.contractalis* is closer to *S.rectacutus* than to *S.brevacutus* based on the relatively long spines on the uncus distally and the densely setose transtilla dorsally. The *praepandalis* group, comprising *S.praepandalis* and *S.curvisetaceus*, can be recognized by the following characters: the bifid uncus with two basally setose teeth; the two needle-shaped setae on the transtilla dorsally; a long, narrowly triangular lobe projecting from the transtilla ventrally; and the long and slender ductus bursae which is about twice as long as the diameter of the corpus bursae. Within the genus, the *praepandalis* group is closer to the *contractalis* group than to the *aureolalis* group.

In this study, four new species are described based on morphological and genetic differences from related species. The morphological differences are given above in the diagnoses of the new species. The genetic distance between species in Lepidoptera are ordinarily greater than 3% (Hebert et al. 2003) in the COI barcode. Among the new species, *S.quadracutus*, *S.curvisetaceus* and *S.brevacutus* are well recognized by distance values greater than 3% from their most closely related species (Table 2). Another new species, *S.rectacutus* showed relatively low genetic distance (2.5%–2.7%) to its most closely related species *S.contractalis*. However, *S.rectacutus* can be distinguished from *S.contractalis* as mentioned above under the diagnosis of *S.rectacutus* and by the key. Moreover, such cases of low genetic divergence are also observed in some other studies in Lepidoptera (Hebert et al. 2003, 2010, Yang et al. 2016). The low interspecific divergence of congeneric species pairs may indicate their recent origin or introgression (Hebert et al. 2003, Zahiri et al. 2014). Based on the covariation between barcodes and morphological traits, *S.rectacutus* is treated as a distinct species.

A relatively high intraspecific divergence was observed in *S.aureolalis* (2.7%). The two specimens concerned, a male and a female, were collected in two localities in Yunnan that are distant by approximately 550 km (Figure 28A). According to the genitalia (Figure 24), the female specimen belongs to the *aureolalis* species group and it can be distinguished from *S.quadracutus* by the two hook-like notches more widely separated from each other. Moreover, no obvious genital variation could be found in the males found in these two localities. Consequently, they are here treated as conspecific. Genital variation is observed in two male specimens collected in Daxichang, Yunnan and Nonggang, Guangxi (genitalia slides no. SYSU0173, SYSU 0909, respectively), both places which are near the north of Vietnam. The distal projection of the sacculus has only one large spine, as in those of *S.quadracutus*, whereas those specimens collected in other places of Yunnan, Thailand and India have two spines. However, other genital traits, as given in the redescription, are all uniform, suggesting their recognition as the same species, *S.aureolalis*.

In the present study, four new species are discovered which are superficially similar to the three described species. Considering the lack of sufficient generic revisions, especially in Oriental region, there is little doubt that many described species have been misplaced and more cryptic species will be revealed within the subfamily. As Munroe (1976a) pointed out, inclusion of genitalia structures and careful analyses of the interspecific and intraspecific differences will certainly help to move ahead to natural classifications as opposed to artificial arrangements. However, the understanding of the phylogenetic relationships between most genera of Pyraustinae is still very imperfect. Phylogenetic systematics based on morphology helps little as pyraustine genera are separated in most cases only by minute morphological differences which are difficult to interpret as apo- or plesiomorphic. The use of genetic data will facilitate species identification and help to understand the interspecific and intergeneric relationships. It calls for more comprehensive investigations on Pyraustinae in the future in order to understand this species-rich subfamily better.

## Supplementary Material

XML Treatment for Spinosuncus

XML Treatment for Spinosuncus contractalis

XML Treatment for Spinosuncus rectacutus

XML Treatment for Spinosuncus brevacutus

XML Treatment for Spinosuncus praepandalis

XML Treatment for Spinosuncus curvisetaceus

XML Treatment for Spinosuncus aureolalis

XML Treatment for Spinosuncus quadracutus

## References

[B1] Bae YS Byun BK Paek MK (2008) Pyralid Moths of Korea (Lepidoptera: Pyraloidea). Korea National Arboretum, 426 pp.

[B2] Bänziger H (1995) *Microstegahomoculorum* sp. n. the most frequently observed lachryphagous moth of man (Lepidoptera, Pyralidae: Pyraustinae). Revue Suisse de Zoologie 102(2): 265–276.

[B3] Butler AG (1881) Descriptions of new Genera and Species of Heterocerous Lepidoptera from Japan. Pyrales and Micros. Transactions of the entomological Society of London 1881: 579–600.

[B4] Caradja A (1925) Ueber Chinas Pyraliden, Tortriciden, Tineiden nebst kurze Betrachtungen, zu denen das Studium dieser Fauna Veranlassung gibt (Eine biogeographische Skizze). Memoriile Sectiunii Stiintifice, Academia Romana seria 3, 3(7): 257–383. [pls 1–2]

[B5] Chen K Zhang DD (2017) Revision of the genus *Pseudopagyda* Slamka, 2013 (Lepidoptera: Pyraloidea: Crambidae: Pyraustinae) with the first reported females. Journal of Environmental Entomology 39(3): 580–587.

[B6] Chen K Zhang DD Stănescu M (2018) Revision of the genus *Eumorphobotys* with descriptions of two new species (Lepidoptera, Crambidae, Pyraustinae). Zootaxa 4472: 489–504. 10.11646/zootaxa.4472.3.4 30313358

[B7] Guenée MA (1854) Deltoïdes et Pyralites. In: Boisduval JBAD de Guenée MA (Eds) Histoire Naturelle des Insectes Species Général des Lépidoptères 8 8.Roret, Paris, 1–448.

[B8] Hampson GF (1893) Moths. The Fauna of British India, including Ceylon and Burma. London Volume 1, 527 pp.

[B9] Hampson GF (1896) Moths. The Fauna of British India, including Ceylon and Burma. London, Volume 4, 594 pp.

[B10] Hampson GF (1898) A revision of the moths of the subfamily Pyraustinae and family Pyralidae. Part I. Proceedings of the General Meetings for Scientific Business of the Zoological Society of London 1898: 590–761. [pls 49–50]

[B11] Hampson GF (1899) A revision of the moths of the subfamily Pyraustinae and family Pyralidae. Part II. Proceedings of the General Meetings for Scientific Business of the Zoological Society of London, 1899: 172–291.

[B12] Hebert PDN Cywinska A Ball SL Dewaard JR (2003) Biological identifications through DNA barcodes. Philosophical Transactions of the Royal Society of London, Series B, Biological Sciences 270: 313–321. 10.1098/rspb.2002.2218 PMC1691236 12614582

[B13] Hebert PDN DeWaard JR Landry JF (2010) DNA barcodes for 1/1000 of the animal kingdom. Biology Letters 6: 359–362. 10.1098/rsbl.2009.0848 20015856 PMC2880045

[B14] Hundsdörfer AK Rubinoff D Attié M Wink M Kitching IJ (2009) A revised molecular phylogeny of the globally distributed hawkmoth genus *Hyles* (Lepidoptera: Sphingidae), based on mitochondrial and nuclear DNA sequences. Molecular Phylogenetics and Evolution 52: 852–865. 10.1016/j.ympev.2009.05.023 19482093

[B15] Inoue H (1982) Pyralidae. In: Inoue H, Sugi S, Kuroko H, Moriuti S, Kawabe A (Eds) Moths of Japan 1+2. Kodansha, Tokyo, 307–404[vol. 1], 223–254 [vol. 2]. [pls. 36–48, 228, 296–314]

[B16] Kirpichnikova VA (1986) Revision of the genus *Paratalanta* Meyr. (LepidopteraPyralidae) of the Far Eastern fauna. In: Ler PA (Ed.) Systematics and ecology of Lepidoptera from the Far East of the USSR.Akademiya Nauk SSSR, Vladivostok, 50–56.

[B17] Kirpichnikova VA (1999) Pyraloidea [sine Phycitinae]. In: Ler PA (Ed.) Key to the insects of Russian Far East 5 (2).Vladivostok, Dalnauka, 320–443.

[B18] Kirpichnikova VA (2009) Pyralids (Lepidoptera, Pyraloidea: Pyralidae, Crambidae) of the fauna of Russian Far East. Vladivostok, Dalnauka, 519 pp.

[B19] Klots AB (1970) Lepidoptera. In: Tuxen SL (Ed.) Taxonomist’s glossary of genitalia in insects.Second revised and enlarged edition. Munksgaard, Copenhagen, Denmark, 115–130.

[B20] Kristensen NP (2003) Skeleton and muscles: adults. In: Kristensen NP (Ed.) Lepidoptera, moths and butterflies.Volume 2: Evolution, systematics, and biogeography. Handbook of Zoology IV (35). Walter de Gruyter, Berlin & New York, 39–131. 10.1515/9783110893724.39

[B21] Lederer J (1863) Beitrag zur Kenntniss der Pyralidinen. Wiener Entomologische Monatschrift 7(8, 10–12): 243–280, 331–504. [pls 242–218]

[B22] Leraut PJA (2012) Moths of Europe, 3, Zygaenids, Pyralides 1 and Brachodids. Rodez, Graphi Imprimeur, 599 pp.

[B23] Li HH Zheng ZM (1996) Methods and techniques of specimens of Microlepidopera. Journal of Shaanxi Normal University (Natural Science Edition) 24(3): 63–70.

[B24] Maes KVN (1994) Some notes on the taxonomic status of the Pyraustinae (sensu Minet 1981 [1982]) and a check list of the Palaearctic Pyraustinae (Lepidoptera, Pyraloidea, Crambidae). Bulletin et Annales de la Société Royale Entomologique de Belgique 130(7–9): 159–168.

[B25] Maes KVN (1995) A comparative morphological study of the adult Crambidae (Lepidoptera, Pyraloidea). Bulletin et Annales de la Société Royale Belge d’Entomologie 131: 383–434.

[B26] Mathew G (2006) An inventory of Indian Pyralids (Lepidoptera: Pyralidae). Zoos’ Print Journal 21(5): 2245–2258. 10.11609/JoTT.ZPJ.667.2245-58

[B27] Meyrick E (1890) On the classification of the Pyralidina of the European fauna. Transactions of the entomological Society of London 1890: 429–492. [pl. 415]

[B28] Munroe EG (1976a) PyraloideaPyralidae comprising the subfamily Pyraustinae tribe Pyraustini (part). In: Dominick RB, et al. (Eds) The Moths of America North of Mexico including Greenland, 13.2A. Classey EW Ltd and The Wedge Entomological Research Foundation, London, 1–78. [pls 1–4]

[B29] Munroe EG (1976b) PyraloideaPyralidae comprising the subfamily Pyraustinae tribe Pyraustini (conclusion). In: Dominick RB, et al. (Eds) The Moths of America North of Mexico including Greenland, 13.2B. Classey EW Ltd and The Wedge Entomological Research Foundation, London, 79–150. [pls 5–9, xiii–xvii]

[B30] Munroe EG (1995) Pyraustinae. In: Heppner JB (Ed.) Atlas of Neotropical Lepidoptera Checklist Part 2: Hyblaeoidea – Pyraloidea – Tortricoidea.Scientific Publishers, Gainesville, Florida, 53–79.

[B31] Munroe EG Mutuura A (1968) Contributions to a study of the Pyraustinae (Lepidoptera: Pyralidae) of temperate East Asia I–IV. The Canadian Entomologist 100(8): 847–868, 100(9): 974–1001.

[B32] Munroe EG Mutuura A (1969) Contributions to a study of the Pyraustinae (Lepidoptera: Pyralidae) of temperate East Asia V–VIII. The Canadian Entomologist 101(3): 299–305, 101(9): 897–906, 101(10): 1069–1077, 101(12): 1239–1248.

[B33] Munroe EG Mutuura A (1970) Contributions to a study of the Pyraustinae (Lepidoptera: Pyralidae) of temperate East Asia IX–X. The Canadian Entomologist 102(3): 294–304, 102(12): 1489–1507.

[B34] Munroe EG Mutuura A (1971) Contributions to a study of the Pyraustinae (Lepidoptera: Pyralidae) of temperate East Asia XI–XII. The Canadian Entomologist 103(2): 173–181, 103(4): 503–506.

[B35] Nuss M Landry B Mally R Vegliante F Trankner A Bauer F Hayden JE Segerer A Schouten R Li H Trofimova T Solis MA De Prins J Speidel W (2003–2018) Global Information System on Pyraloidea http://www.pyraloidea.org

[B36] Posada D (2008) jModelTest: phylogenetic model averaging. Molecular Biology and Evolution 25(7): 1253–1256. 10.1093/molbev/msn083 18397919

[B37] Regier JC Mitter C Solis MA Hayden JE Landry B Nuss M Simonsen TJ Yen S-H Zwick A Cummings MP (2012) A molecular phylogeny for the pyraloid moths (Lepidoptera: Pyraloidea) and its implications for higher-level classification. Systematic Entomology 37(4): 635–656. 10.1111/j.1365-3113.2012.00641.x

[B38] Robinson GS (1976) The preparation of slides of Lepidoptera genitalia with special reference to the Microlepidoptera. Entomologist’s Gazette 27: 127–132.

[B39] Ronquist F Teslenko M van der Mark P Ayres DL Darling A Höhna S Larget B Liu L Suchard MA Huelsenbeck JP (2012) MrBayes 3.2: efficient Bayesian phylogenetic inference and model choice across a large model space. Systematic Biology 61: 539–542. 10.1093/sysbio/sys029 22357727 PMC3329765

[B40] Scholtens BG Solis AM (2015) Annotated check list of the Pyraloidea (Lepidoptera) of America North of Mexico. ZooKeys 535: 1–136. 10.3897/zookeys.535.6086 PMC4669914 26668552

[B41] Shaffer JC Munroe EG (2007) Crambidae of Aldabra Atoll (Lepidoptera: Pyraloidea). Tropical Lepidoptera 14[2003](1–2): 1–110.

[B42] Shaffer M Nielsen ES Horak M (1996) Pyraloidea. In: Nielsen ES Edwards ED Rangsi TV (Eds) Checklist of the Lepidoptera of Australia.In: Nielsen ES. Monographs on Australian Lepidoptera 4 4. CSIRO Division of Entomology, Canberra, 164–199.

[B43] Shibuya J (1928) The systematic study on the formosan Pyralidae. Journal of the Faculty of Agriculture, Hokkaido Imperial University 22(1): 1–300. [pls. 1–9]

[B44] Shibuya J (1929) On the known and unrecorded species of the Japanese Pyraustinae (Lepid.). Journal of the Faculty of Agriculture, Hokkaido Imperial University 25: 151–242.

[B45] Simon C Buckley TR Frati F Stewart JB Beckenbach AT (2006) Incorporating molecular evolution into phylogenetic analysis, and a new compilation of conserved polymerase chain reaction primers for animal mitochondrial DNA. Annual Review of Ecology, Evolution, and Systematics 37: 545–579. 10.1146/annurev.ecolsys.37.091305.110018

[B46] Slamka F (2013) Pyraloidea of Europe 3, Pyraustinae and Spilomelinae. Bratislava, 357 pp.

[B47] Snellen PCT (1890) A catalogue of the Pyralidina of Sikkim collected by Henry J. Elwes and the late Otto Möller, with notes by H. J. Elwes. Transactions of the entomological Society of London: 557–647. [pls 519–520]

[B48] Solis MA Maes KVN (2002) Preliminary phylogenetic analysis of the subfamilies of Crambidae (PyraloideaLepidoptera). Belgian Journal of Entomology 4: 53–95.

[B49] Speidel W (1996) Pyraloidea [part]. In: Karsholt O Razowski J (Eds) The Lepidoptera of Europe.A distributional checklist. Apollo Books, Stenstrup, 166–183, 187–196, 319–327.

[B50] Stamatakis A (2014) RAxML version 8: a tool for phylogenetic analysis and post-analysis of large phylogenies. Bioinformatics 30: 1312–1313. 10.1093/bioinformatics/btu033 24451623 PMC3998144

[B51] Swinhoe C (1901) New genera and species of Eastern and Australian moths. Annals and Magazine of Natural History, including Zoology, Botany and Geology (ser. 7) 8: 16–27.

[B52] Tamura K Stecher G Peterson D Filipski A Kumar S (2013) MEGA6: Molecular Evolutionary Genetics Analysis version 6.0. Molecular biology and evolution 30: 2725–2729. 10.1093/molbev/mst197 24132122 PMC3840312

[B53] Thompson JD Higgins DG Gibson TJ (1994) CLUSTAL W: improving the sensitivity of progressive multiple sequence alignment through sequence weighting, position-specific gap penalties and weight matrix choice. Nucleic acids research 22: 4673–4680. 10.1093/nar/22.22.4673 7984417 PMC308517

[B54] Tränkner A Li HH Nuss M (2009) On the systematics of *Anania* Hübner, 1823 (Pyraloidea: Crambidae: Pyraustinae). Nota lepidopterologica 32(1): 63–80.

[B55] Wahlberg N Wheat CW (2008) Genomic outposts serve the phylogenomic pioneers: designing novel nuclear markers for genomic DNA extractions of Lepidoptera. Systematic Biology 57(2): 231–242. 10.1080/10635150802033006 18398768

[B56] Wang PY (1980) Lepidoptera. Pyralidae. Economic Insect Fauna of China. Science Press, Beijing, 229 pp.

[B57] Wang HY Speidel W (2000) Pyraloidea (Pyralidae, Crambidae). Guide Book to Insects in Taiwan. Shu Shin Books, Taipei, 295 pp.

[B58] Warren W (1896) New species of Pyralidae from the Khasia Hills. Annals and Magazine of Natural History, including Zoology, Botany and Geology (ser. 6) 18: 107–119, 163–177, 214–232.

[B59] Yamanaka H Yoshiyasu Y Sasaki A (2013) Pyraloidea. In: Nasu Y Hirowatari T Kishida Y (Eds) The Standard of Moths in Japan IV.Gakken Education Publishing, Tokyo, 60–84, 314–478.

[B60] Yang Z Landry J-F Hebert PDN (2016) A DNA barcode library for North American Pyraustinae (Lepidoptera: Pyraloidea: Crambidae). PLoS ONE 11(10): e0161449. 10.1371/journal.pone.0161449 PMC5063472 27736878

[B61] Zahiri R Lafontaine JD Schmidt BC deWaard JR Zakharov EV Hebert PDN (2014) A transcontinental challenge – A test of DNA barcode performance for 1,541 species of Canadian Noctuoidea (Lepidoptera). PLoS ONE 9(3): e92797. 10.1371/journal.pone.0092797 PMC3965468 24667847

[B62] Zhang DD (2003) A taxonomic study on the tribe Pyraustini from the Mainland of China (Lepidoptera: Crambidae: Pyraustinae). PhD Thesis, Tianjin, China: Nankai University.

[B63] Zhang DD Cai YP Li HH (2014) Taxonomic review of the genus *Paratalanta* Meyrick, 1890 (Lepidoptera: Crambidae: Pyraustinae) from China, with descriptions of two new species. Zootaxa 3753(2): 118–132. 24872284 10.11646/zootaxa.3753.2.2

